# Supplementary material for: 1H-Imidazole-2,5-Dicarboxamides as NS4A Peptidomimetics: Identification of a New Approach to Inhibit HCV-NS3 Protease
Source: Biomolecules. 2020 Mar 21;10(3):479. doi: 10.3390/biom10030479 (PMC7175367; doi:10.3390/biom10030479)
Supplement: Supplementary file 1 [file biomolecules-10-00479-s001.pdf]

1H-Imidazole-2,5-dicarboxamides as NS4A Peptidomimetics: Identification of a New Venue to Inhibit HCV-NS3 Protease

Abdelsattar M. Omar[a,b], Mahmoud A. El-Faky[c], Stefan T. Arold[d], Sameh H. Soror[e,f], Maan T. Khayat[a], Hani Z. Asfour[g], Faida H. Bamane[h] and Moustafa E. El-Araby[a]*

[a] *Department of Pharmaceutical Chemistry, Faculty of Pharmacy, King Abdulaziz University, Alsulaymanyah, Jeddah 21589, Saudi Arabia*

[b] *Department of Pharmaceutical Chemistry, Faculty of Pharmacy, Al-Azhar University, Cairo, Egypt*

[c] *Department of Natural Products and Alternative Medicine, Faculty of Pharmacy, King Abdulaziz University, Alsulaymanyah, Jeddah 21589, Saudi Arabia*

[d] *Computational Bioscience Research Center, Division of Biological and Environmental Sciences and Engineering, King Abdullah University of Science and Technology, Thuwal, Saudi Arabia*

[e] *Center for Scientific Excellence Helwan Structural Biology Research (HSBR), Faculty of Pharmacy, Helwan University, Ain Helwan, P.O. 11795, Cairo, Egypt*

[f] *Department of Biochemistry and Molecular Biology, Faculty of Pharmacy, Helwan University, Ain Helwan, P.O. 11795, Cairo, Egypt*

[g] *Department of Medical Microbiology and Parasitology, Faculty of Medicine, King Abdulaziz University, Jeddah 21589, Saudi Arabia*

[h] *Department of Biochemistry, Faculty of Medicine, King Abdulaziz University, Jeddah 21589, Saudi Arabia*

S1. Superposition of NS4A in four different crystal structures

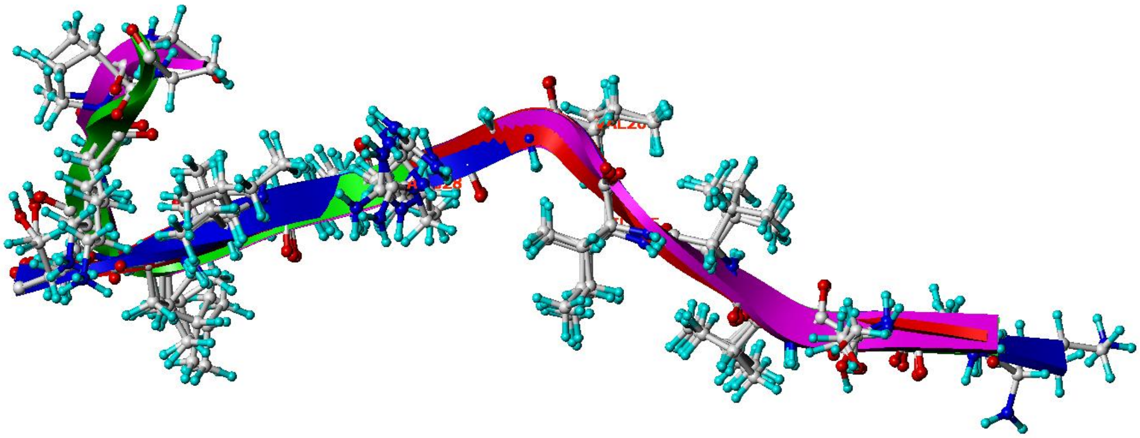

Figure S1-A: Conserved conformation of NS4A peptide in bound form with NS3 protease domain. Examples were downloaded from PDB website (rcsb.org) and illustrated as follows: 1A1R (Green), 2OC1 (magenta), 3OYP (Red) and 4U01 (Blue)

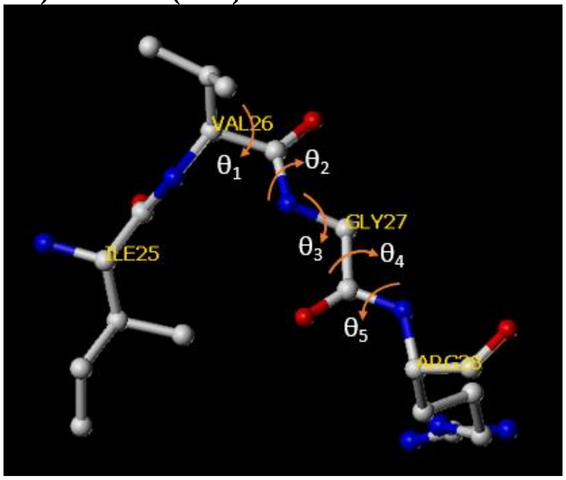

Figure S1-B. Dihedral angles θ_n of the bound NS4A's planar region (PDB Code: 1NS3). Numerical values of θ_1 to θ_5 are listed in Table S1-A.

Torsion	Actual	Deviation from Plane
θ_1	13.9	+13.9 (eclipsed cis)
θ_2	179.4	-0.6
θ_3	184.6	+4.6
θ_4	191.6	+11.6
θ_5	184.3	+4.3

Table S1. Dihedral angles of core part of bound NS4A (PDB Code: 1NS3).

S2. Spectra of Intermediates and Final Compounds

5-(tert-butyl) 2-ethyl 1H-imidazole-2,5-dicarboxylate (2)

This report was created by ACD/NMR Processor Academic Edition. For more information go to www.acdlabs.com/nmrproc/

3/2/2017 1:46:23 PM

Formula	$C_{11}H_{16}N_2O_4$	FW	240.2557

Acquisition Time (sec)	2.6477	Comment	CIX-1078756, KAU1501KTM-156_1, 1H, DMSO-d6 @ 211492			Date	10 Sep 2015 20:25:04
Date Stamp	10 Sep 2015 20:25:04						
File Name	C:\Users\mtk0005\Google Drive\KAU\MEl-Araby\12-BIO3193-03 Imidazole Cpds\1H-NMR\KAU1501KTM-156_1\fid					Frequency (MHz)	300.13
Nucleus	1H	Number of Transients	8	Origin	spect	Original Points Count	16384
Owner	Administrator	Points Count	16384	Pulse Sequence	zg	Receiver Gain	256.00
SW(cyclical) (Hz)	6188.12	Solvent	DMSO-d6	Spectrum Offset (Hz)	1853.6917	Spectrum Type	STANDARD
Sweep Width (Hz)	6187.74	Temperature (degree C)	26.160				

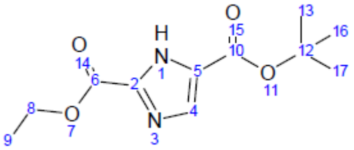

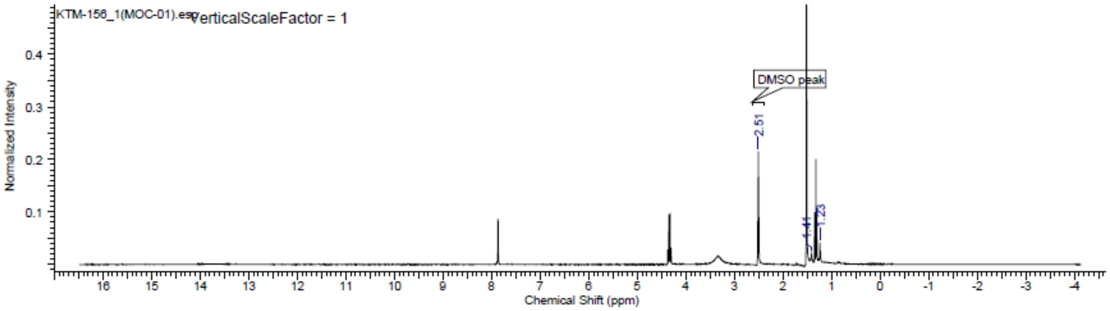

Peak ID	Time	Mass Found
3	1.41	240.11

3:(Time: 1.41) 1.7e+007

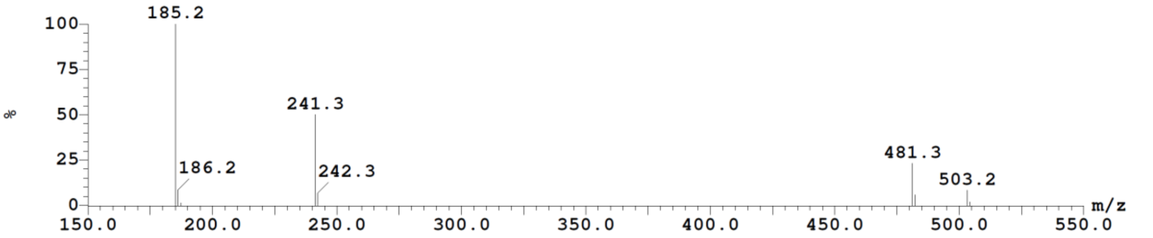

This report was created by ACD/NMR Processor Academic Edition. For more information go to www.acdlabs.com/nmrproc/

3/2/2017 1:50:15 PM

Acquisition Time (sec)	5.3084	*Comment*	CIX-1078848, KAU1501KTM-161_1, 1H, DMSO-d6 @ 211890			*Date*	14 Sep 2015 21:24:48
Date Stamp	14 Sep 2015 21:24:48						
File Name	C:\Users\mtk0005\Google Drive\KAU\MEI-Araby\12-BIO3193-03 Imidazole Cpds\1H-NMR\KAU1501KTM-161_1\fid					*Frequency (MHz)*	300.13
Nucleus	1H	*Number of Transients*	16	*Origin*	spect	*Original Points Count*	32768
Owner	guest	*Points Count*	32768	*Pulse Sequence*	zg30	*Receiver Gain*	645.10
SW(cyclical) (Hz)	6172.84	*Solvent*	DMSO-d6	*Spectrum Offset (Hz)*	1853.4263	*Spectrum Type*	STANDARD
Sweep Width (Hz)	6172.65	*Temperature (degree C)*	26.400				

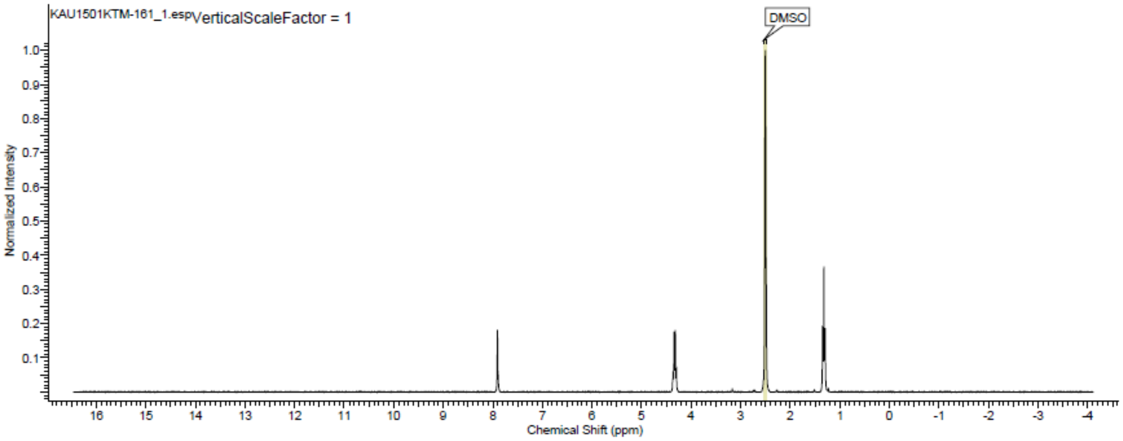

Peak ID	Time	Mass Found
6	1.55	184.05

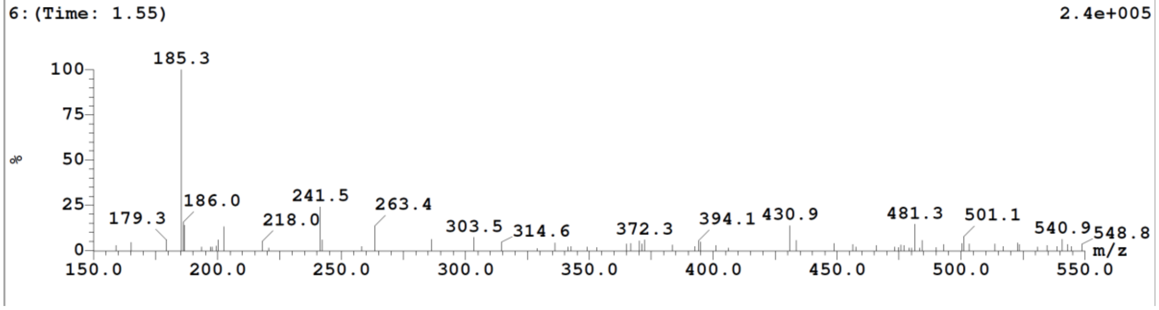

Ethyl 4-((2-(methylamino)-2-oxoethyl)carbamoyl)-1H-imidazole-2-carboxylate (4a)

This report was created by ACD/NMR Processor Academic Edition. For more information go to www.acdlabs.com/nmrproc/

3/6/2017 3:58:45 PM

Formula	$C_{10}H_{14}N_4O_4$	FW	254.2426

Acquisition Time (sec)	5.3084	Comment	CIX-1079327, KAU1501KTM-190_1, 1H, DMSO-d6 @ 216449			Date	13 Oct 2015 20:31:28
Date Stamp	13 Oct 2015 20:31:28						
File Name	C:\Users\mtk0005\Google Drive\KAU\MEI-Araby\12-BIO3193-03 Imidazole Cpds\1H-NMR\KAU1501KTM-190_1\fid					Frequency (MHz)	300.13
Nucleus	1H	Number of Transients	16	Origin	spect	Original Points Count	32768
Owner	guest	Points Count	32768	Pulse Sequence	zg30	Receiver Gain	512.00
SW(cyclical) (Hz)	6172.84	Solvent	DMSO-d6	Spectrum Offset (Hz)	1853.4263	Spectrum Type	STANDARD
Sweep Width (Hz)	6172.65	Temperature (degree C)	26.900				

^{1}H NMR (300 MHz, DMSO-d_6) δ 8.19 (br. s., 1H), 7.81 (s, 2H), 4.35 (q, J = 7.16 Hz, 2H), 3.81 (d, J = 5.65 Hz, 2H), 2.59 (d, J = 4.52 Hz, 3H), 1.33 (t, J = 7.16 Hz, 3H)

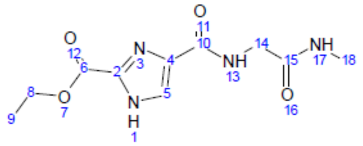

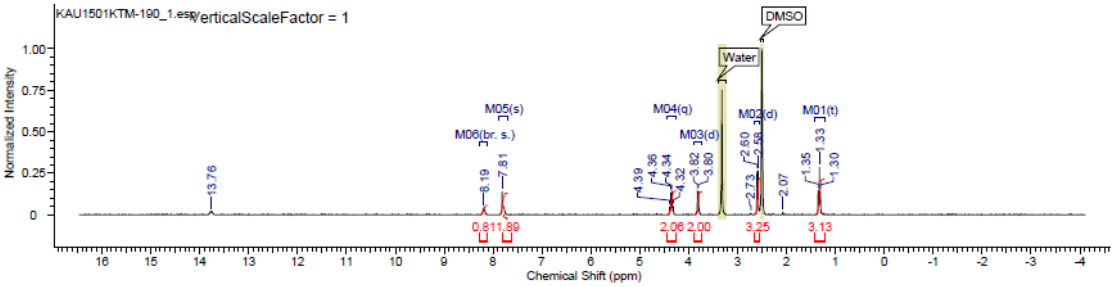

Peak ID	Time	Mass Found
16	1.57	254.10

16: (Time: 1.58) 3.3e+005

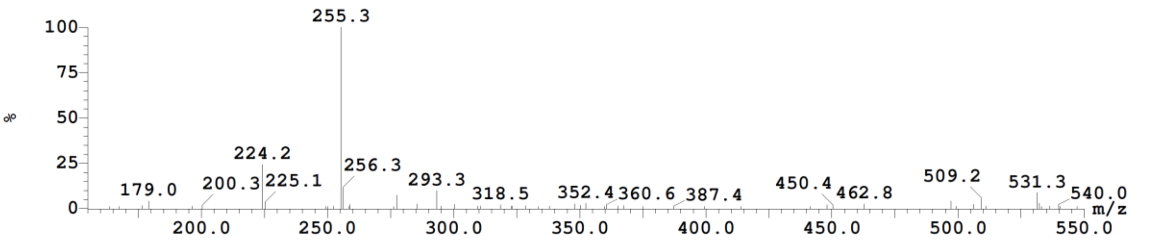

Ethyl 5-((pyridin-2-ylmethyl)carbamoyl)-1H-imidazole-2-carboxylate (4c)

This report was created by ACD/NMR Processor Academic Edition. For more information go to www.acdlabs.com/nmrproc/

3/2/2017 2:00:53 PM

Acquisition Time (sec)	5.3084	***Comment***	CIX-1079258, KAU1501KTM-185_1, 1H, DMSO-d6 @ 215311			***Date***	06 Oct 2015 20:50:40
Date Stamp	06 Oct 2015 20:50:40						
File Name	C:\Users\mtk0005\Google Drive\KAU\MEI-Araby\12-BIO3193-03 Imidazole Cpds\1H-NMR\KAU1501KTM-185_1\fid					***Frequency (MHz)***	300.13
Nucleus	1H	***Number of Transients***	16	***Origin***	spect	***Original Points Count***	32768
Owner	guest	***Points Count***	32768	***Pulse Sequence***	zg30	***Receiver Gain***	456.10
SW(cyclical) (Hz)	6172.84	***Solvent***	DMSO-d6	***Spectrum Offset (Hz)***	1853.4263	***Spectrum Type***	STANDARD
Sweep Width (Hz)	6172.65	***Temperature (degree C)***	25.500				

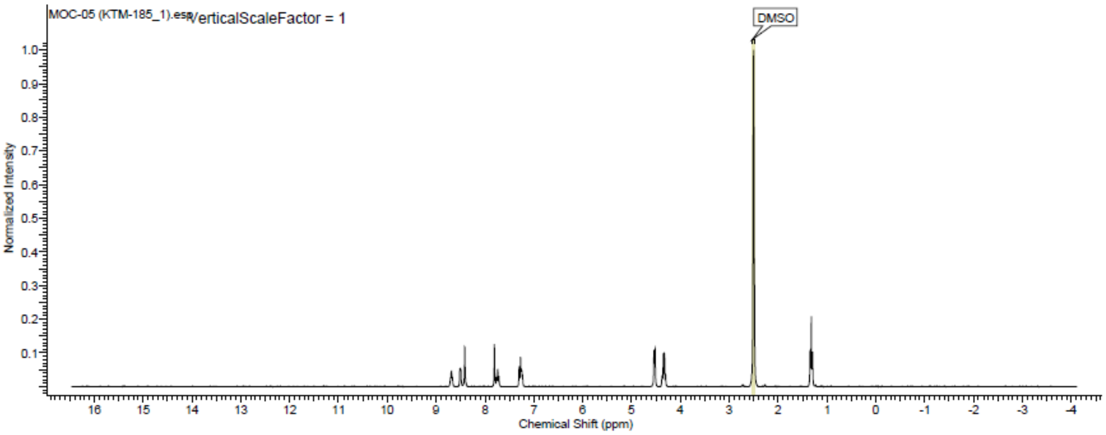

MOC-11

This report was created by ACD/NMR Processor Academic Edition. For more information go to www.acdlabs.com/nmrproc/

5/6/2017 10:03:53 AM

Acquisition Time (sec)	2.6477	***Comment***	CIX-1082893, KAU1501KTM-228_1A, 1H, CD3OD - Methanol-D4 @ 225918				
Date	16 Nov 2015 23:17:36			***Date Stamp***	16 Nov 2015 23:17:36		
File Name	C:\Users\Maankhayat\Google Drive\KAU\MEI-Araby\12-BIO3193-03 Imidazole Cpds\1H-NMR\KAU1501KTM-228_1A\fid					***Frequency (MHz)***	300.13
Nucleus	1H	***Number of Transients***	8	***Origin***	spect	***Original Points Count***	16384
Owner	Administrator	***Points Count***	16384	***Pulse Sequence***	zg	***Receiver Gain***	645.10
SW(cyclical) (Hz)	6188.12	***Solvent***	METHANOL-d4	***Spectrum Offset (Hz)***	1853.6917	***Spectrum Type***	STANDARD
Sweep Width (Hz)	6187.74	***Temperature (degree C)***	25.160				

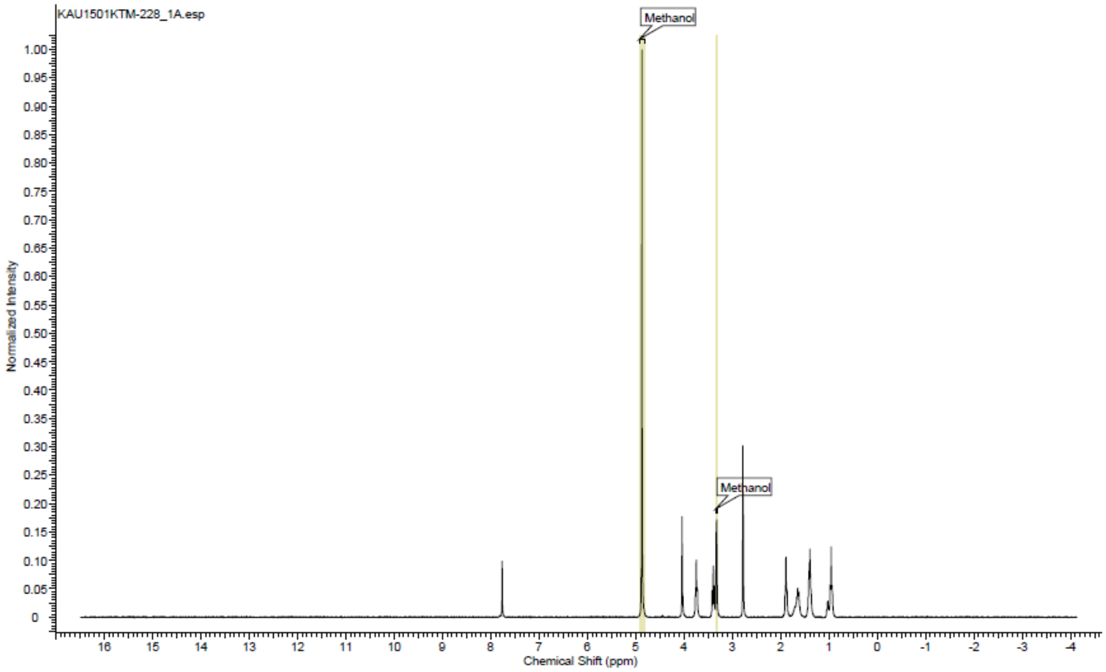

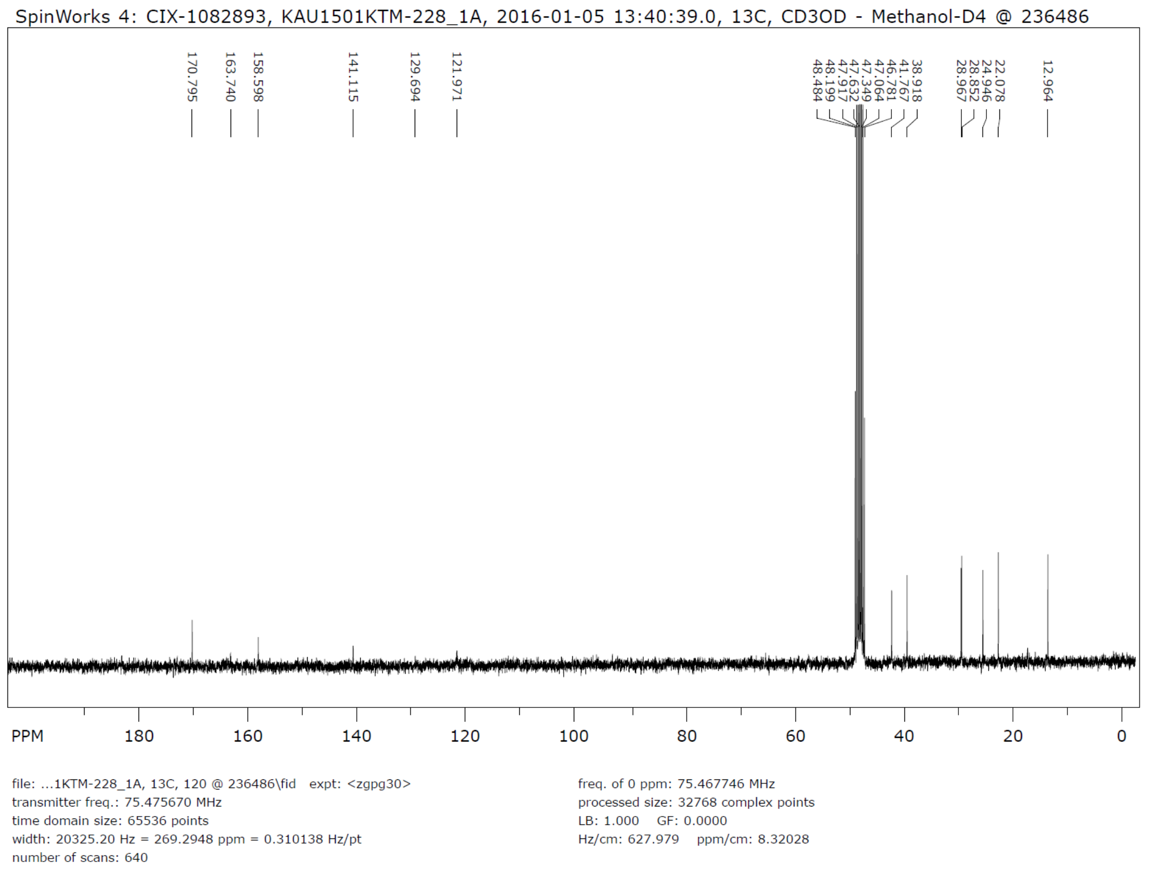
SpinWorks 4: CIX-1082893, KAU1501KTM-228_1A, 2016-01-05 13:40:39.0, 13C, CD3OD - Methanol-D4 @ 236486
170.795
163.740
158.598
141.115
129.694
121.971
48.484
48.199
47.917
47.632
47.349
47.064
46.781
41.767
38.918
28.967
28.852
24.946
22.078
12.964
PPM 180 160 140 120 100 80 60 40 20 0
file: ...1KTM-228_1A, 13C, 120 @ 236486\fid expt: <zgpg30>
transmitter freq.: 75.475670 MHz
time domain size: 65536 points
width: 20325.20 Hz = 269.2948 ppm = 0.310138 Hz/pt
number of scans: 640
freq. of 0 ppm: 75.467746 MHz
processed size: 32768 complex points
LB: 1.000 GF: 0.0000
Hz/cm: 627.979 ppm/cm: 8.32028

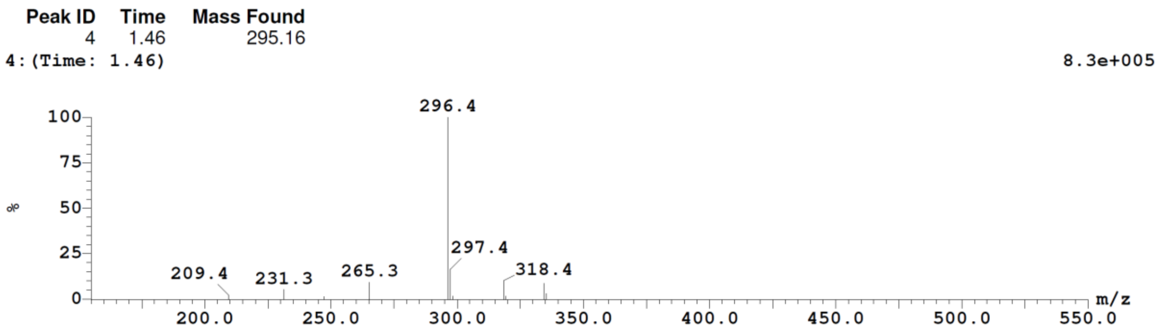
Peak ID Time Mass Found
4 1.46 295.16
4: (Time: 1.46)
8.3e+005
100
75
%
50
25
0
296.4
297.4
209.4
231.3
265.3
318.4
200.0 250.0 300.0 350.0 400.0 450.0 500.0 550.0
m/z

MOC-34

This report was created by ACD/NMR Processor Academic Edition. For more information go to www.acdlabs.com/nmrproc/

5/8/2017 8:03:26 AM

Formula $C_{15}H_{18}N_6O_3$	FW 330.3418

Acquisition Time (sec)	2.6477	Comment	CIX-1080569, KAU1501KTM-212_1A, 1H, CD3OD - Methanol-D4 @ 219296				
Date	22 Oct 2015 11:42:24			Date Stamp	22 Oct 2015 11:42:24		
File Name	C:\Users\mtk0005\Google Drive\KAU\MEI-Araby\12-BIO3193-03 Imidazole Cpds\1H-NMR\KAU1501KTM-212_1A\fid					Frequency (MHz)	300.13
Nucleus	1H	Number of Transients	8	Origin	spect	Original Points Count	16384
Owner	Administrator	Points Count	16384	Pulse Sequence	zg	Receiver Gain	812.70
SW(cyclical) (Hz)	6188.12	Solvent	METHANOL-d4	Spectrum Offset (Hz)	1853.6917	Spectrum Type	STANDARD
Sweep Width (Hz)	6187.74	Temperature (degree C)	25.160				

1H NMR (300 MHz, METHANOL-d_4) δ 8.65 (d, J = 4.91 Hz, 1H), 8.08 - 8.32 (m, 1H), 7.69 - 7.93 (m, 2H), 7.54 - 7.69 (m, 1H), 3.47 (dd, J = 5.85, 19.83 Hz, 4H), 1.97 (s, 3H), 0.92 - 1.19 (m, 1H)

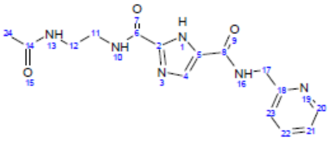

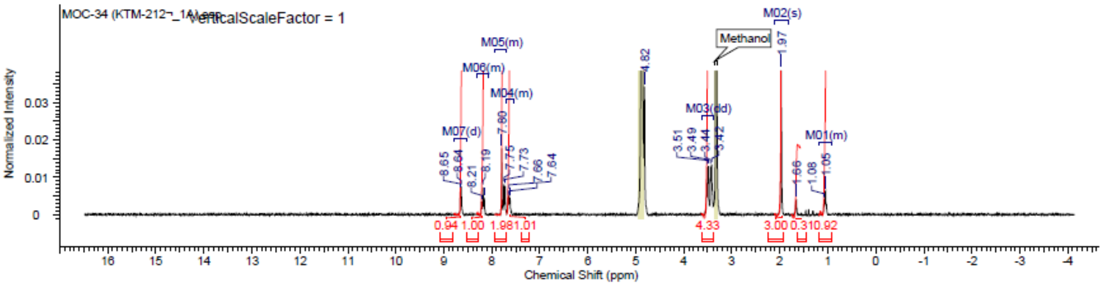

SpinWorks 4: CIX-1080569, KAU1501KTM-212_1A, 2016-01-05 13:40:39.0, 13C, CD3OD - Methanol-D4 @ 236474

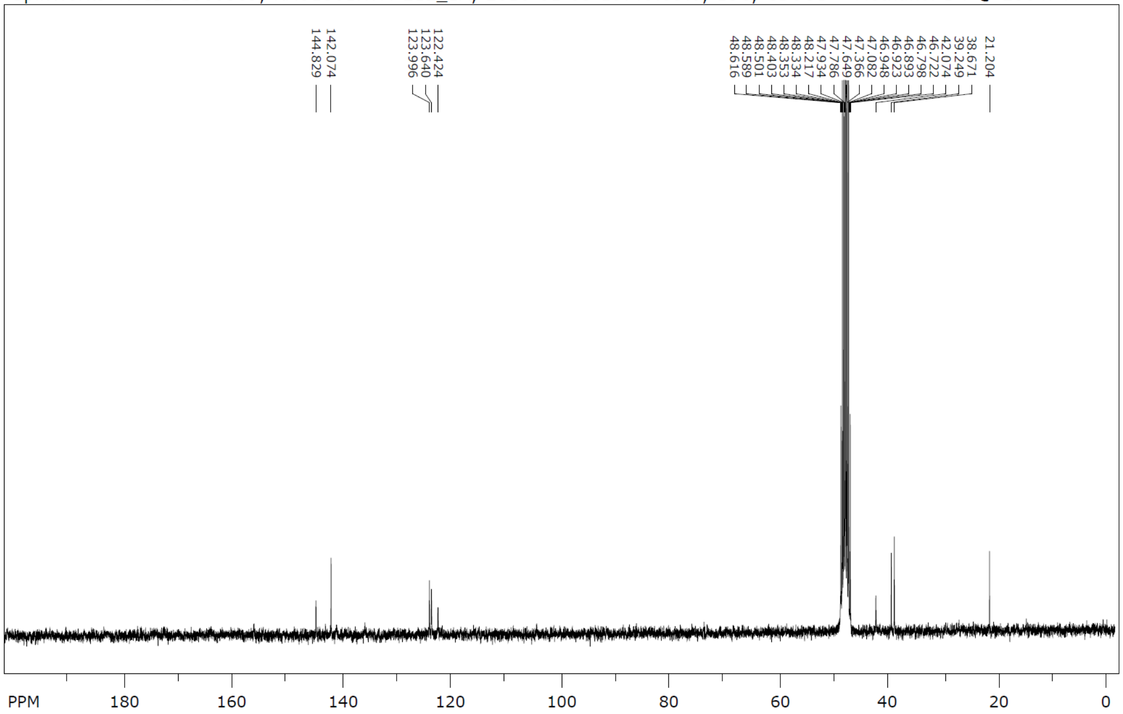

file: ...1KTM-212_1A, 13C, 110 @ 236474\fid   expt: <zgpg30>
transmitter freq.: 75.475670 MHz
time domain size: 65536 points
width: 20325.20 Hz = 269.2948 ppm = 0.310138 Hz/pt
number of scans: 640

freq. of 0 ppm: 75.467746 MHz
processed size: 32768 complex points
LB: 1.000   GF: 0.0000
Hz/cm: 615.363   ppm/cm: 8.15313

Peak ID	Time	Mass Found
8	3.18	330.14

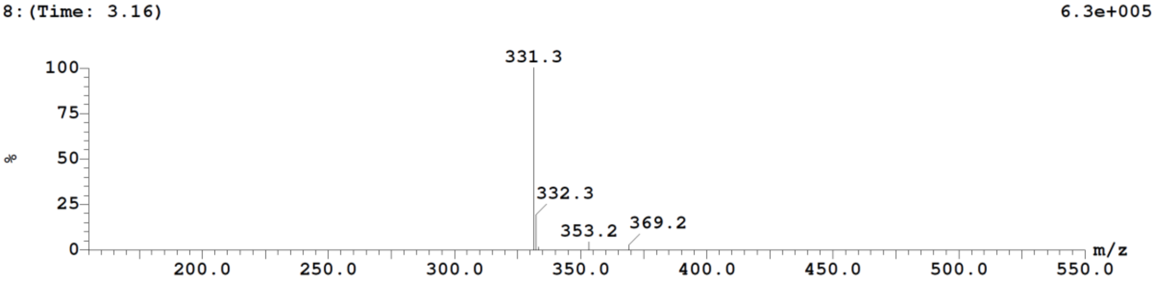

MOC-23

This report was created by ACD/NMR Processor Academic Edition. For more information go to www.acdlabs.com/nmrproc/

5/9/2017 1:06:20 PM

**Acquisition Time (sec)**	5.3084	**Comment**	CIX-1084257, KAU1501KTM-249_1, 1H, CD3OD - Methanol-D4 @ 229355				
**Date**	26 Nov 2015 20:52:32			**Date Stamp**	26 Nov 2015 20:52:32		
**File Name**	C:\Users\mtk0005\Google Drive\KAU\MEI-Araby\12-BIO3193-03 Imidazole Cpds\1H-NMR\KAU1501KTM-249_1\fid					**Frequency (MHz)**	300.13
**Nucleus**	1H	**Number of Transients**	16	**Origin**	spect	**Original Points Count**	32768
**Owner**	guest	**Points Count**	32768	**Pulse Sequence**	zg30	**Receiver Gain**	912.30
**SW(cyclical) (Hz)**	6172.84	**Solvent**	METHANOL-d4	**Spectrum Offset (Hz)**	1853.4263	**Spectrum Type**	STANDARD
**Sweep Width (Hz)**	6172.65	**Temperature (degree C)**	26.100				

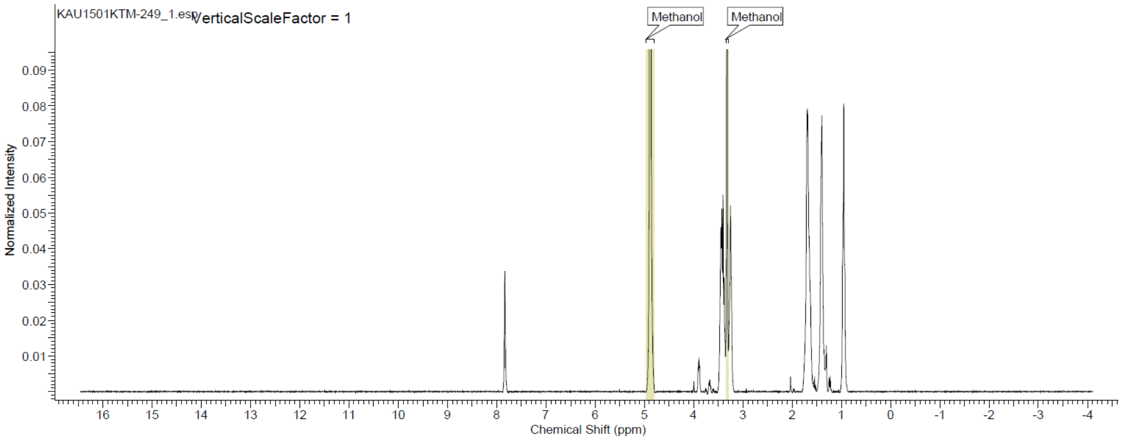

MOC-24

This report was created by ACD/NMR Processor Academic Edition. For more information go to www.acdlabs.com/nmrproc/

5/9/2017 1:02:51 PM

**Acquisition Time (sec)**	5.3084	**Comment**	CIX-1084297, KAU1501KTM-250_1, 2015-12-10 15:21:28.0, 1H, CD3OD - Methanol-D4 @ 234378				
**Date**	10 Dec 2015 20:50:24	**Date Stamp**	10 Dec 2015 20:50:24				
**File Name**	C:\Users\mtk0005\Google Drive\KAU\MEI-Araby\12-BIO3193-03 Imidazole Cpds\1H-NMR\KAU1501KTM-250_1\fid					**Frequency (MHz)**	300.13
**Nucleus**	1H	**Number of Transients**	16	**Origin**	spect	**Original Points Count**	32768
**Owner**	guest	**Points Count**	32768	**Pulse Sequence**	zg30	**Receiver Gain**	574.70
**SW(cyclical) (Hz)**	6172.84	**Solvent**	METHANOL-d4	**Spectrum Offset (Hz)**	1853.4263	**Spectrum Type**	STANDARD
**Sweep Width (Hz)**	6172.65	**Temperature (degree C)**	26.900				

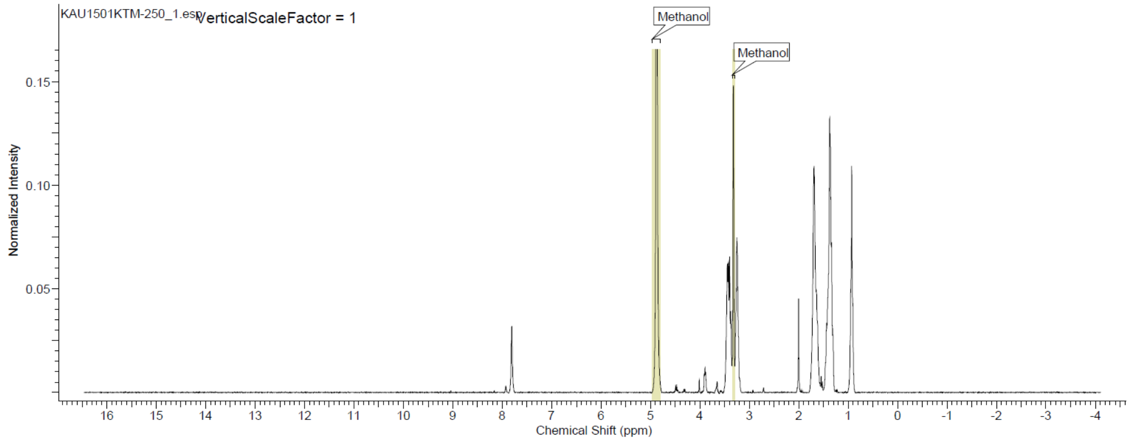

SpinWorks 4: CIX-1084297, KAU1501KTM-250_1, 2016-01-05 13:40:39.0, 13C, CD3OD - Methanol-D4 @ 236488

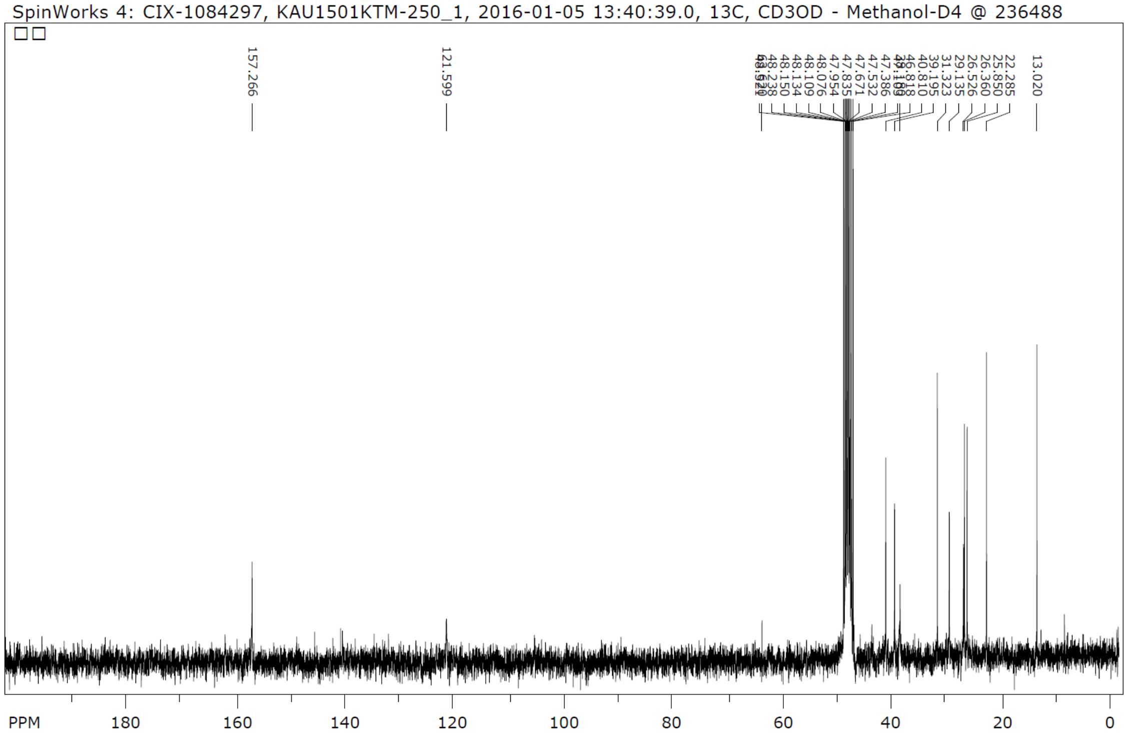

file: ...01KTM-250_1, 13C, 240 @ 236488\fid expt: <zgpg30>
transmitter freq.: 75.475670 MHz
time domain size: 65536 points
width: 20325.20 Hz = 269.2948 ppm = 0.310138 Hz/pt
number of scans: 640

freq. of 0 ppm: 75.467746 MHz
processed size: 32768 complex points
LB: 1.000 GF: 0.0000
Hz/cm: 618.225 ppm/cm: 8.19105

Peak ID	Time	Mass Found
10	1.84	351.24

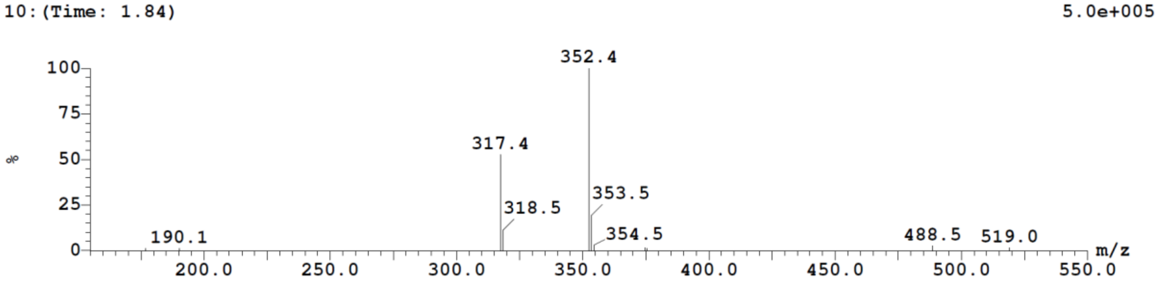

MOC-26

This report was created by ACD/NMR Processor Academic Edition. For more information go to www.acdlabs.com/nmrproc/

5/9/2017 12:55:16 PM

Acquisition Time (sec)	5.3084	***Comment***	CIX-1084300, KAU1501KTM-253_1, 2015-12-10 15:21:28.0, 1H, CD3OD - Methanol-D4 @ 234380				
Date	10 Dec 2015 21:01:04	***Date Stamp***	10 Dec 2015 21:01:04				
File Name	C:\Users\mtk0005\Google Drive\KAU\MEI-Araby\12-BIO3193-03 Imidazole Cpds\1H-NMR\KAU1501KTM-253_1\fid					***Frequency (MHz)***	300.13
Nucleus	1H	***Number of Transients***	16	***Origin***	spect	***Original Points Count***	32768
Owner	guest	***Points Count***	32768	***Pulse Sequence***	zg30	***Receiver Gain***	912.30
SW(cyclical) (Hz)	6172.84	***Solvent***	METHANOL-d4	***Spectrum Offset (Hz)***	1853.4263	***Spectrum Type***	STANDARD
Sweep Width (Hz)	6172.65	***Temperature (degree C)***	27.000				

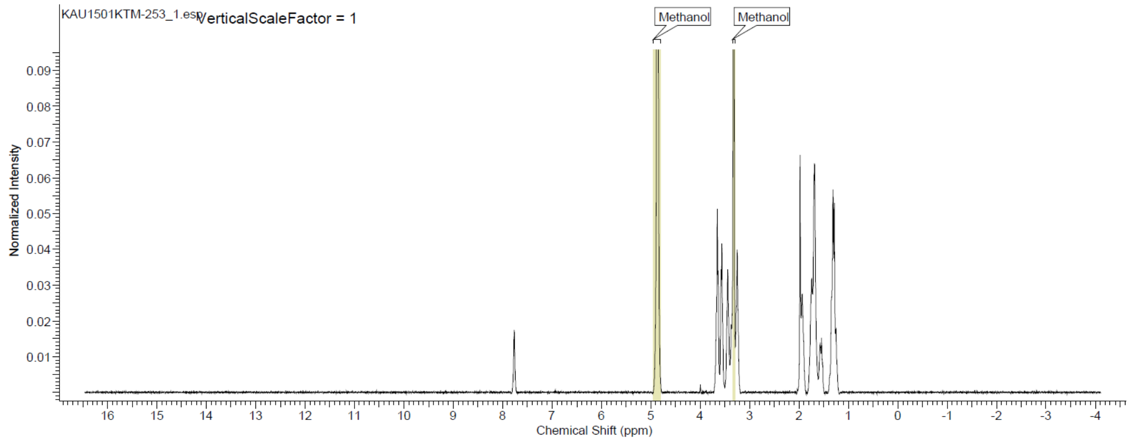

MOC-27

This report was created by ACD/NMR Processor Academic Edition. For more information go to www.acdlabs.com/nmrproc/

5/9/2017 12:48:13 PM

Acquisition Time (sec)	5.3084	***Comment***	CIX-1086662, KAU1501KTM-254_1, 1H, CD3OD - Methanol-D4 @ 231795				
Date	03 Dec 2015 21:26:40			***Date Stamp***	03 Dec 2015 21:26:40		
File Name	C:\Users\mtk0005\Google Drive\KAU\MEI-Araby\12-BIO3193-03 Imidazole Cpds\1H-NMR\KAU1501KTM-254_1\fid					***Frequency (MHz)***	300.13
Nucleus	1H	***Number of Transients***	16	***Origin***	spect	***Original Points Count***	32768
Owner	guest	***Points Count***	32768	***Pulse Sequence***	zg30	***Receiver Gain***	724.10
SW(cyclical) (Hz)	6172.84	***Solvent***	METHANOL-d4	***Spectrum Offset (Hz)***	1853.4263	***Spectrum Type***	STANDARD
Sweep Width (Hz)	6172.65	***Temperature (degree C)***	26.800				

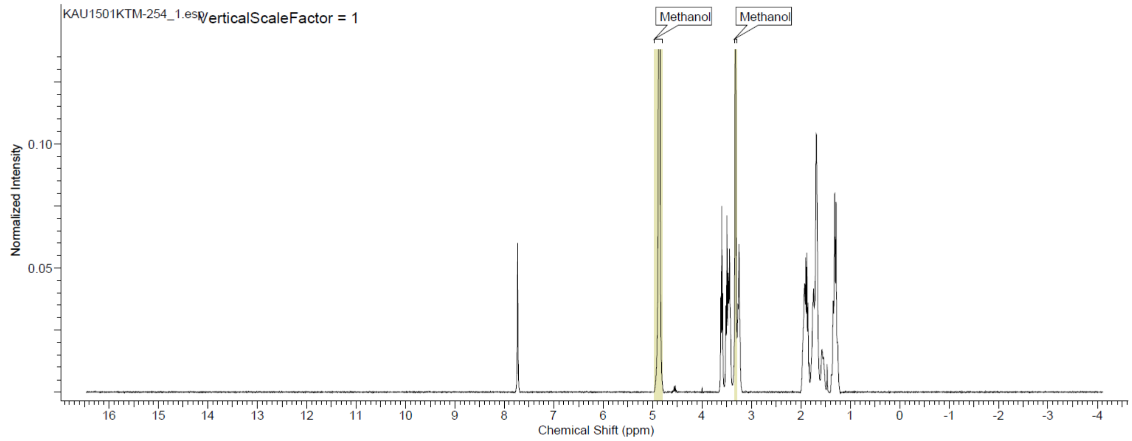

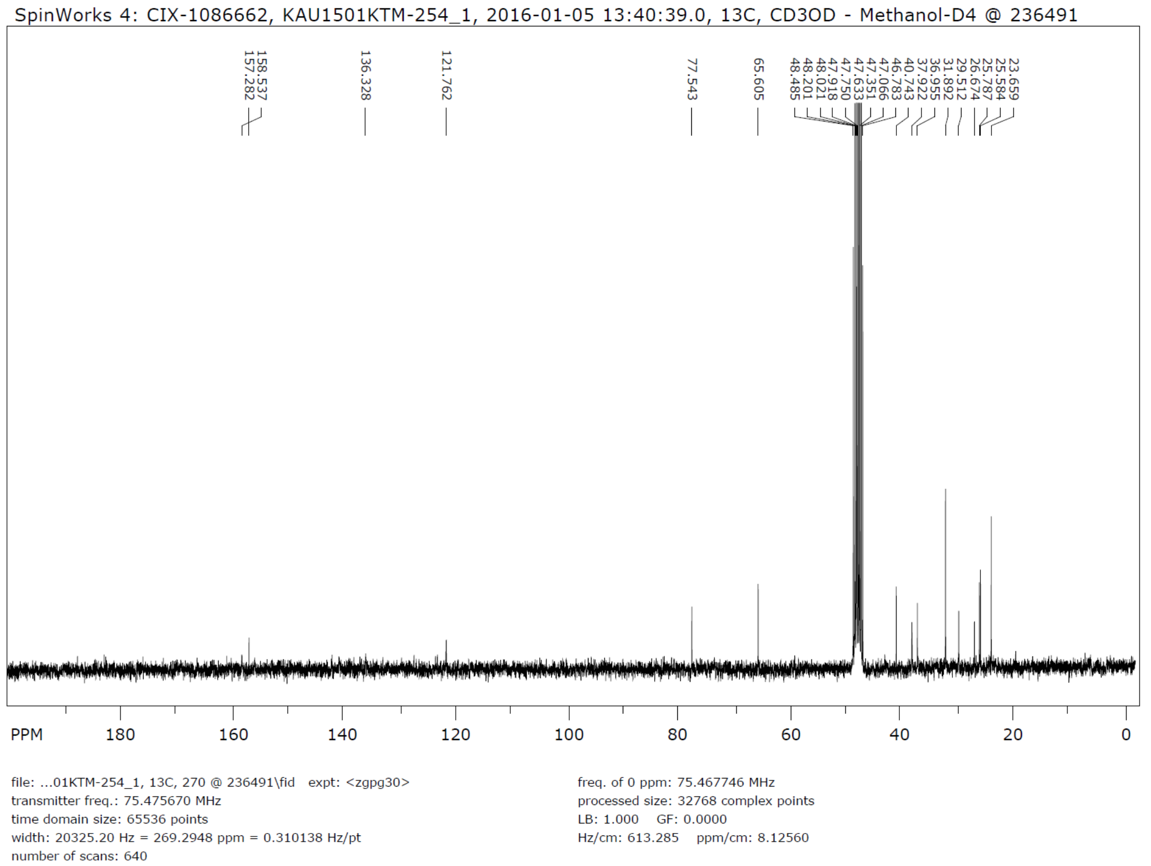

Peak ID	Time	Mass Found
14	2.01	407.26

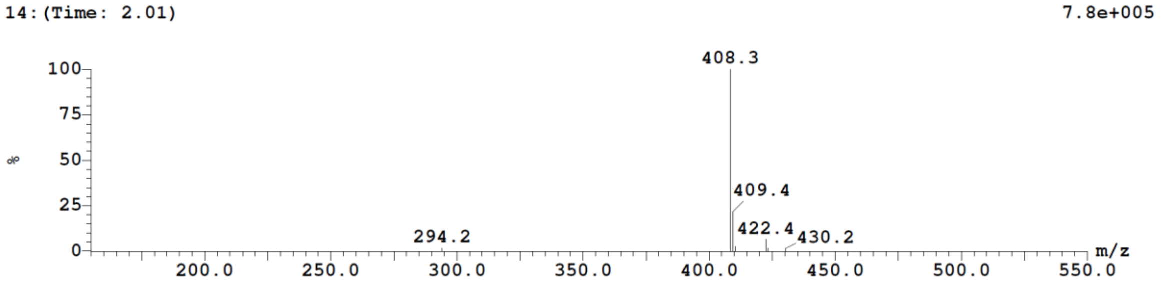

MOC-28

This report was created by ACD/NMR Processor Academic Edition. For more information go to www.acdlabs.com/nmrproc/

5/9/2017 1:10:35 PM

**Acquisition Time (sec)**	5.3084	**Comment**	CIX-1084301, KAU1501KTM-311_1, 2015-12-21 15:41:06.0, 1H, CD3OD - Methanol-D4 @ 236170				
**Date**	21 Dec 2015 22:39:12	**Date Stamp**	21 Dec 2015 22:39:12				
**File Name**	C:\Users\mtk0005\Google Drive\KAU\MEl-Araby\12-BIO3193-03 Imidazole Cpds\1H-NMR\KAU1501KTM-311_1\fid					**Frequency (MHz)**	300.13
**Nucleus**	1H	**Number of Transients**	16	**Origin**	spect	**Original Points Count**	32768
**Owner**	guest	**Points Count**	32768	**Pulse Sequence**	zg30	**Receiver Gain**	512.00
**SW(cyclical) (Hz)**	6172.84	**Solvent**	METHANOL-d4	**Spectrum Offset (Hz)**	1853.4263	**Spectrum Type**	STANDARD
**Sweep Width (Hz)**	6172.65						

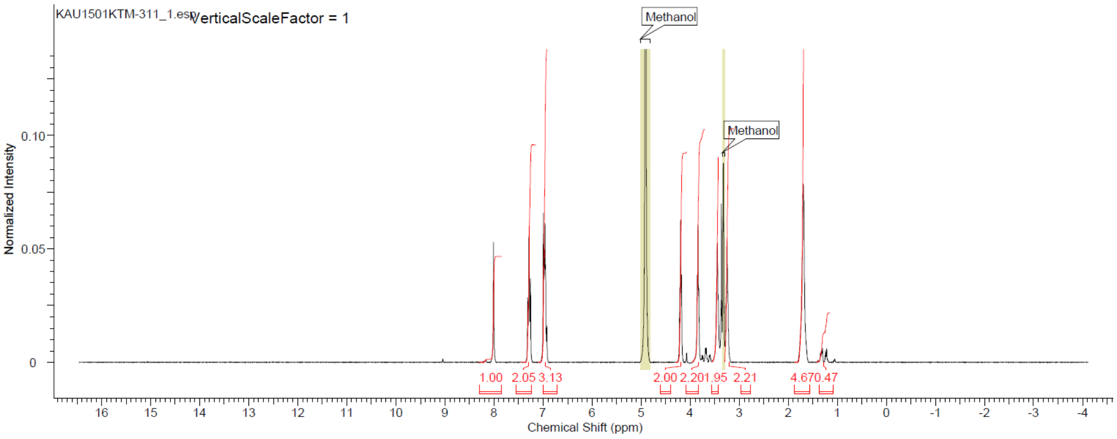

SpinWorks 4: CIX-1084301, KAU1501KTM-311_1, 2015-12-21 15:41:06.0, 13C, CD3OD - Methanol-D4 @ 236170

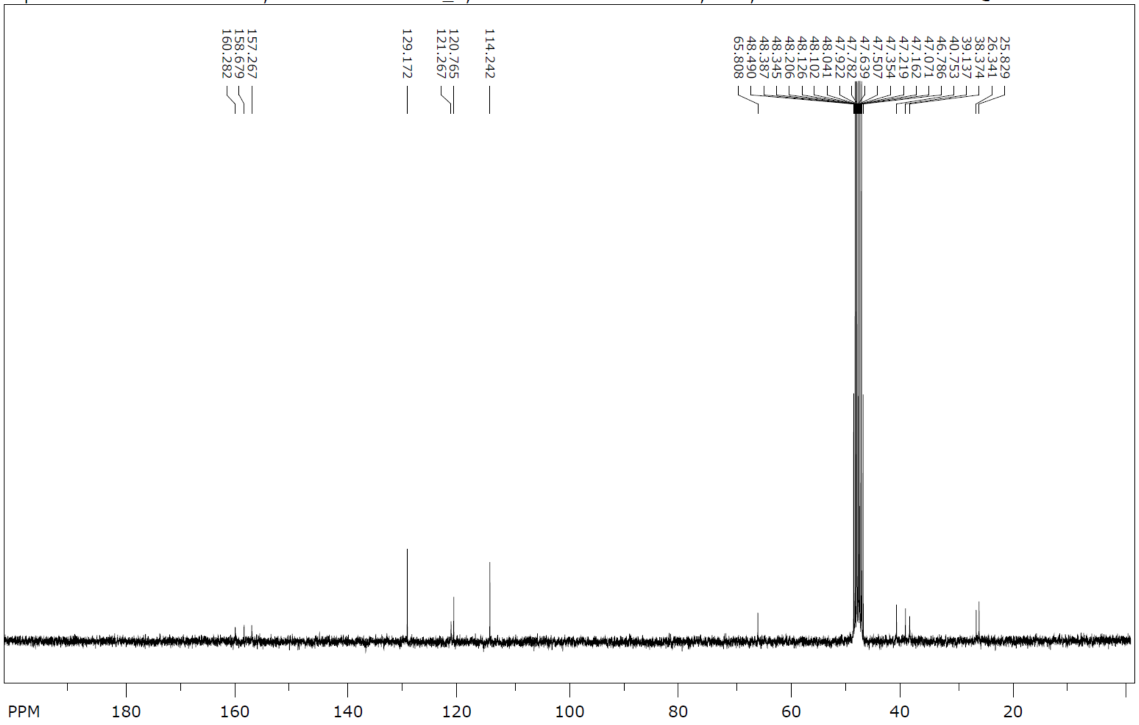

file: ...KTM-311_1, 13C, 28931 @ 236170\fid expt: <zgpg30>
transmitter freq.: 75.475670 MHz
time domain size: 65536 points
width: 20325.20 Hz = 269.2948 ppm = 0.310138 Hz/pt
number of scans: 600

freq. of 0 ppm: 75.467746 MHz
processed size: 32768 complex points
LB: 1.000 GF: 0.0000
Hz/cm: 614.662 ppm/cm: 8.14385

Peak ID	Time	Mass Found
3	1.86	387.20

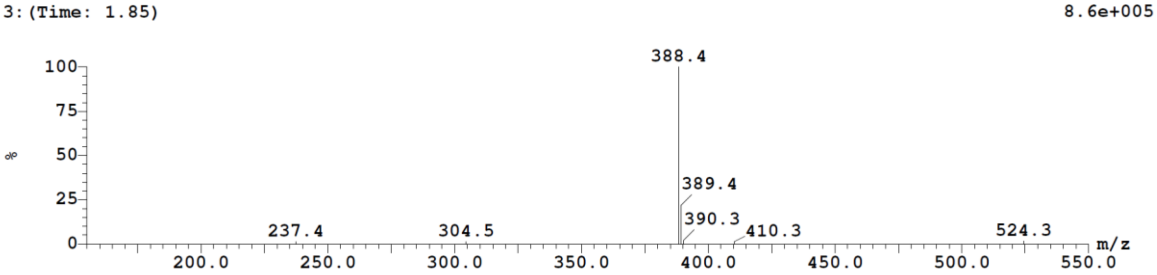

MOC-29

This report was created by ACD/NMR Processor Academic Edition. For more information go to www.acdlabs.com/nmrproc/

5/9/2017 9:06:02 AM

Acquisition Time (sec)	5.3084	***Comment***	CIX-1086663, KAU1501KTM-255_1, 1H, CD3OD - Methanol-D4 @ 231796				
Date	03 Dec 2015 22:07:12			***Date Stamp***	03 Dec 2015 22:07:12		
File Name	C:\Users\mtk0005\Google Drive\KAU\MEl-Araby\12-BIO3193-03 Imidazole Cpds\1H-NMR\KAU1501KTM-255_1\fid					***Frequency (MHz)***	300.13
Nucleus	1H	***Number of Transients***	16	***Origin***	spect	***Original Points Count***	32768
Owner	guest	***Points Count***	32768	***Pulse Sequence***	zg30	***Receiver Gain***	812.70
SW(cyclical) (Hz)	6172.84	***Solvent***	METHANOL-d4	***Spectrum Offset (Hz)***	1853.4263	***Spectrum Type***	STANDARD
Sweep Width (Hz)	6172.65	***Temperature (degree C)***	26.600				

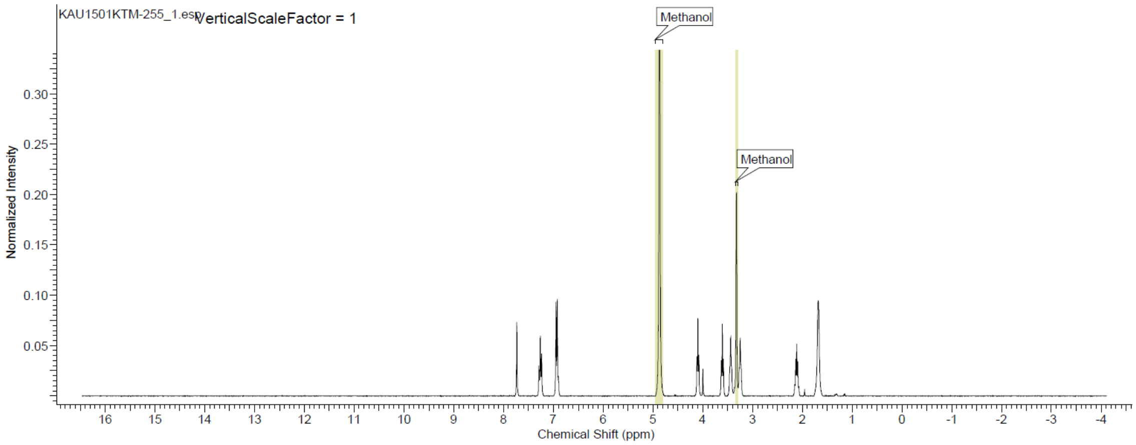

SpinWorks 4: CIX-1086663, KAU1501KTM-255_1, 2016-01-05 13:40:39.0, 13C, CD3OD - Methanol-D4 @ 236492

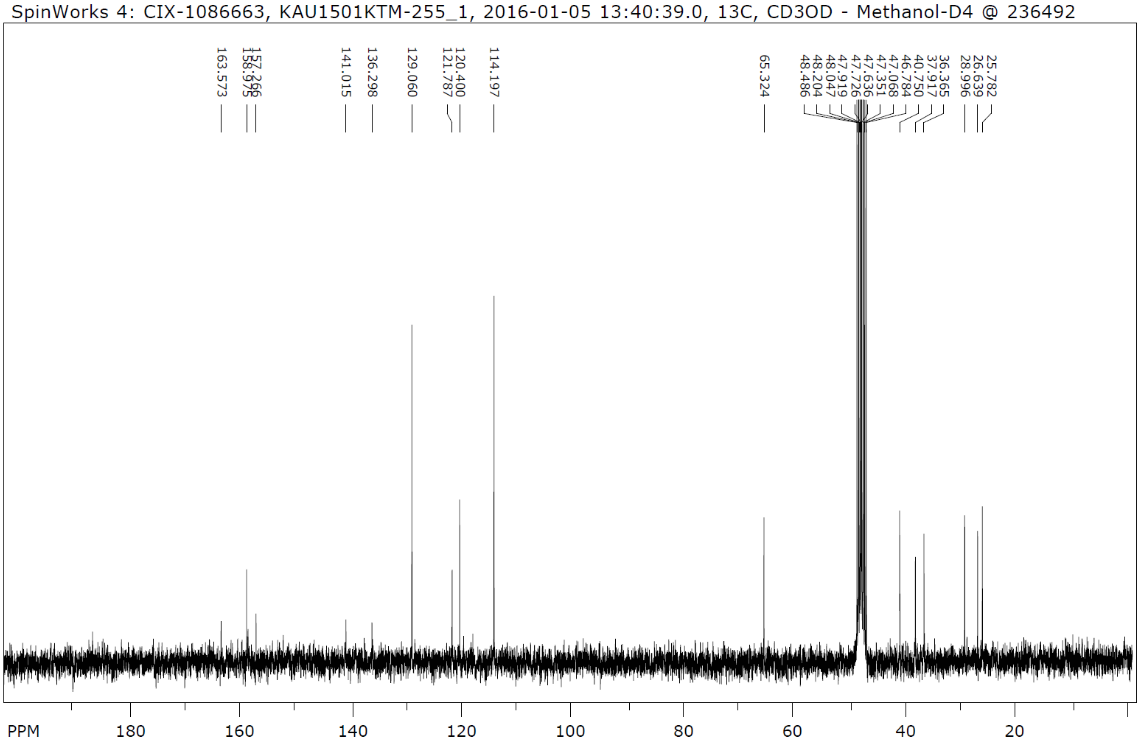

file: ...01KTM-255_1, 13C, 280 @ 236492\fid expt: <zgpg30>
transmitter freq.: 75.475670 MHz
time domain size: 65536 points
width: 20325.20 Hz = 269.2948 ppm = 0.310138 Hz/pt
number of scans: 640

freq. of 0 ppm: 75.467746 MHz
processed size: 32768 complex points
LB: 1.000 GF: 0.0000
Hz/cm: 616.813 ppm/cm: 8.17235

Peak ID	Time	Mass Found
12	1.89	401.22

12: (Time: 1.89) 7.5e+005

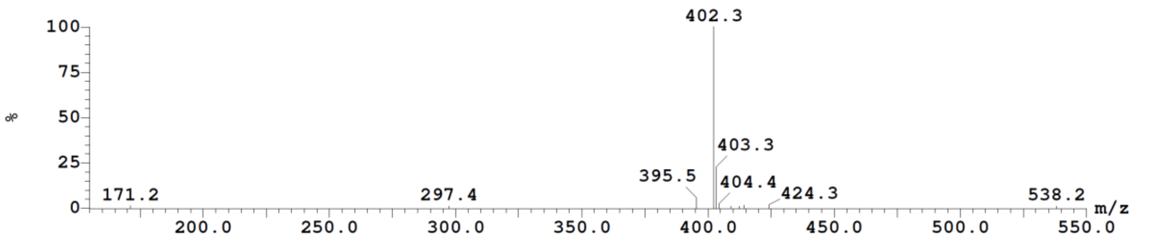

Peak ID	Time	Mass Found
18	2.87	352.20

18: (Time: 2.87) 7.6e+005

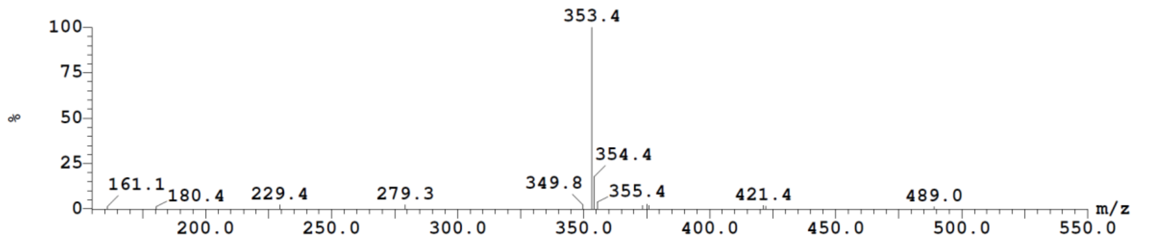

MOC-30

This report was created by ACD/NMR Processor Academic Edition. For more information go to www.acdlabs.com/nmrproc/

5/9/2017 8:57:15 AM

**Acquisition Time (sec)**	5.3084	**Comment**	CIX-1086665, KAU1501KTM-257_1, 1H, CD3OD - Methanol-D4 @ 231798				
**Date**	03 Dec 2015 21:37:20			**Date Stamp**	03 Dec 2015 21:37:20		
**File Name**	C:\Users\mtk0005\Google Drive\KAU\MEI-Araby\12-BIO3193-03 Imidazole Cpds\1H-NMR\KAU1501KTM-257_1\fid					**Frequency (MHz)**	300.13
**Nucleus**	1H	**Number of Transients**	16	**Origin**	spect	**Original Points Count**	32768
**Owner**	guest	**Points Count**	32768	**Pulse Sequence**	zg30	**Receiver Gain**	812.70
**SW(cyclical) (Hz)**	6172.84	**Solvent**	METHANOL-d4	**Spectrum Offset (Hz)**	1853.4263	**Spectrum Type**	STANDARD
**Sweep Width (Hz)**	6172.65	**Temperature (degree C)**	26.600				

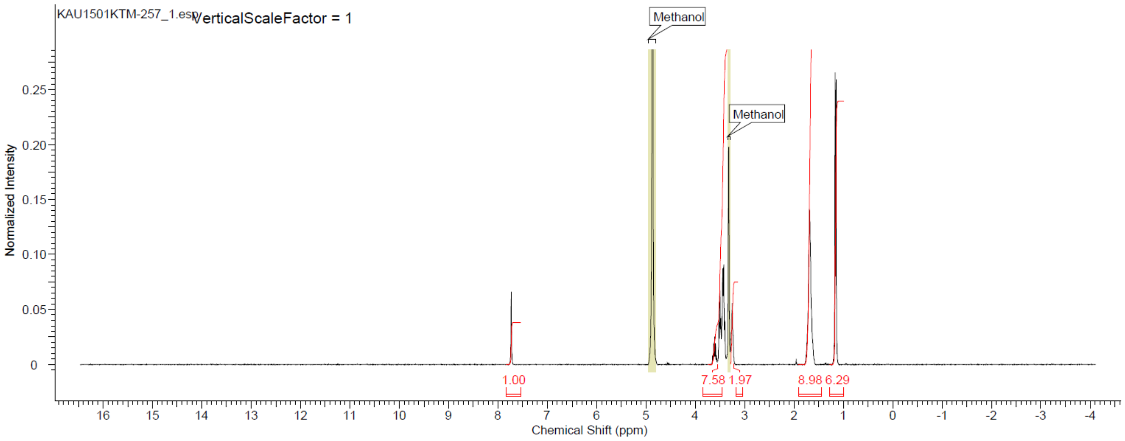

SpinWorks 4: CIX-1086665, KAU1501KTM-257_1, 2016-01-05 13:40:39.0, 13C, CD3OD - Methanol-D4 @ 236494

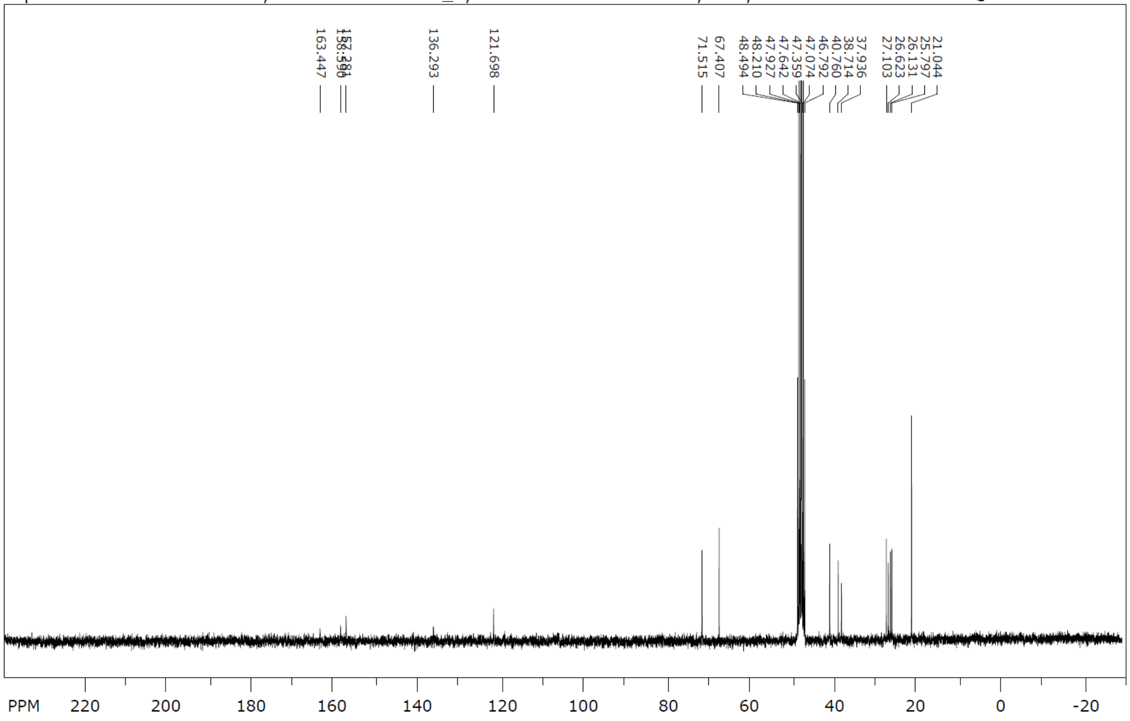

file: ...01KTM-257_1, 13C, 300 @ 236494\fid expt: <zgpg30>
transmitter freq.: 75.475670 MHz
time domain size: 65536 points
width: 20325.20 Hz = 269.2948 ppm = 0.310138 Hz/pt
number of scans: 640

freq. of 0 ppm: 75.467746 MHz
processed size: 32768 complex points
LB: 1.000 GF: 0.0000
Hz/cm: 813.008 ppm/cm: 10.77179

Peak ID	Time	Mass Found
15	2.08	381.25

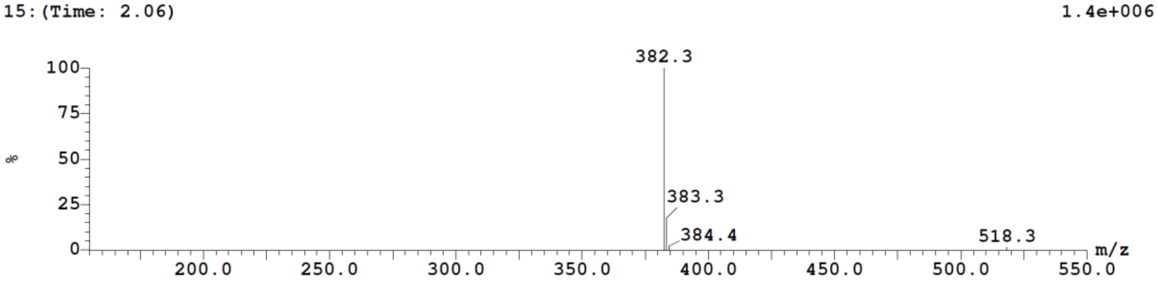

MOC-31

This report was created by ACD/NMR Processor Academic Edition. For more information go to www.acdlabs.com/nmrproc/

5/9/2017 8:44:35 AM

Acquisition Time (sec)	5.3084	***Comment***	CIX-1086666, KAU1501KTM-258_1, 1H, CD3OD - Methanol-D4 @ 231799				
Date	03 Dec 2015 21:43:44			***Date Stamp***	03 Dec 2015 21:43:44		
File Name	C:\Users\mtk0005\Google Drive\KAU\MEI-Araby\12-BIO3193-03 Imidazole Cpds\1H-NMR\KAU1501KTM-258_1\fid					***Frequency (MHz)***	300.13
Nucleus	1H	***Number of Transients***	16	***Origin***	spect	***Original Points Count***	32768
Owner	guest	***Points Count***	32768	***Pulse Sequence***	zg30	***Receiver Gain***	724.10
SW(cyclical) (Hz)	6172.84	***Solvent***	METHANOL-d4	***Spectrum Offset (Hz)***	1853.4263	***Spectrum Type***	STANDARD
Sweep Width (Hz)	6172.65	***Temperature (degree C)***	26.700				

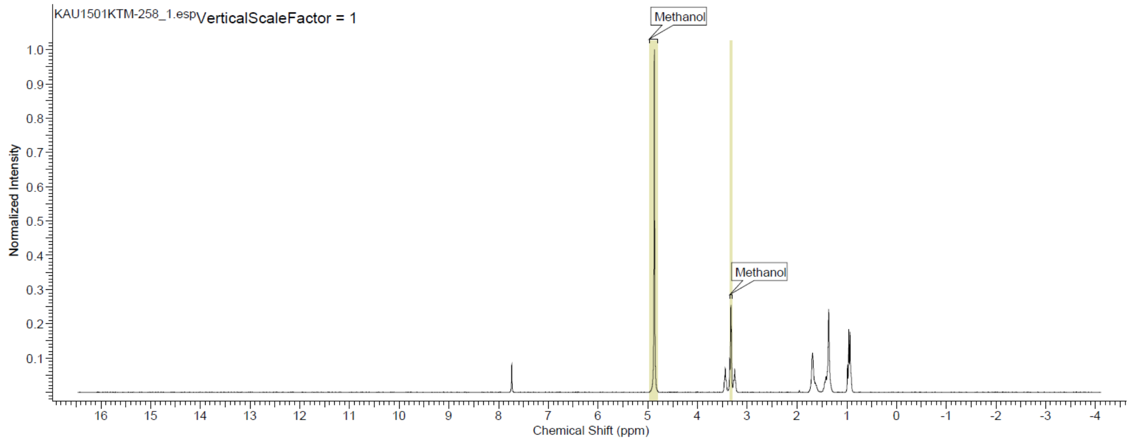

SpinWorks 4:

CIX-1086666, KAU1501KTM-258_1, 2016-01-05 13:40:39.0, 13C, CD3OD - Methanol-D4 @ 236495

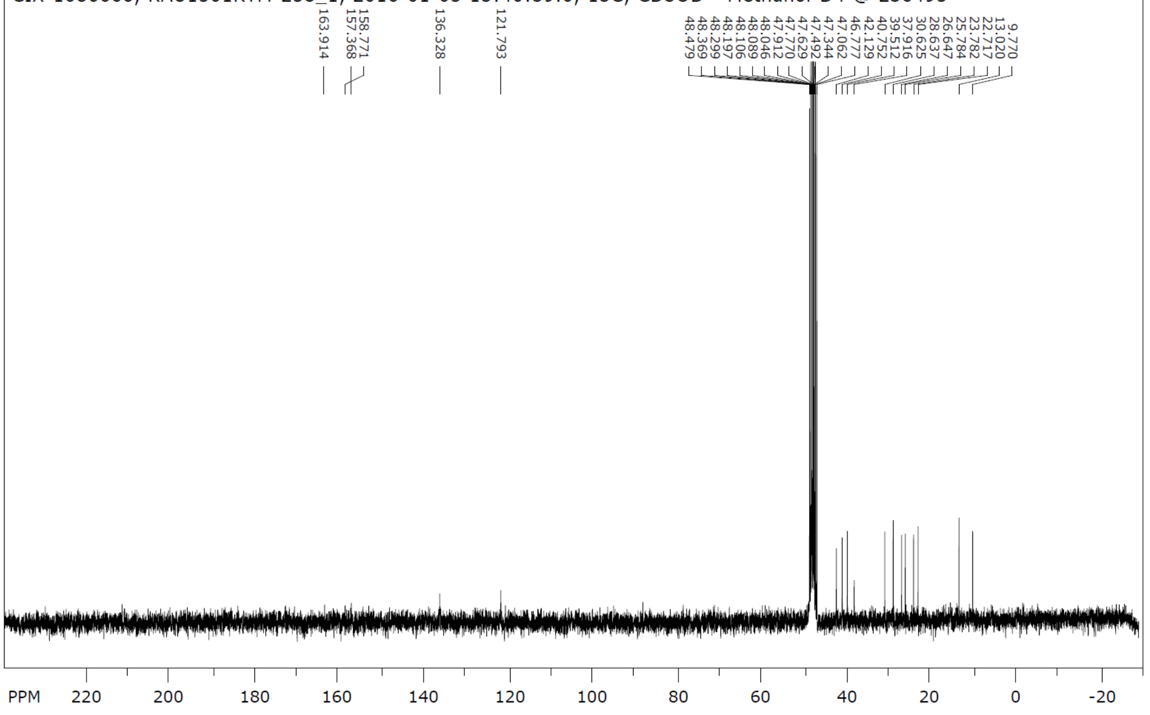

file: ...01KTM-258_1, 13C, 310 @ 236495\fid expt: <zgpg30>
transmitter freq.: 75.475670 MHz
time domain size: 65536 points
width: 20325.20 Hz = 269.2948 ppm = 0.310138 Hz/pt
number of scans: 640

freq. of 0 ppm: 75.467746 MHz
processed size: 32768 complex points
LB: 1.000 GF: 0.0000
Hz/cm: 813.008 ppm/cm: 10.77179

Peak ID	Time	Mass Found
2	1.19	379.27

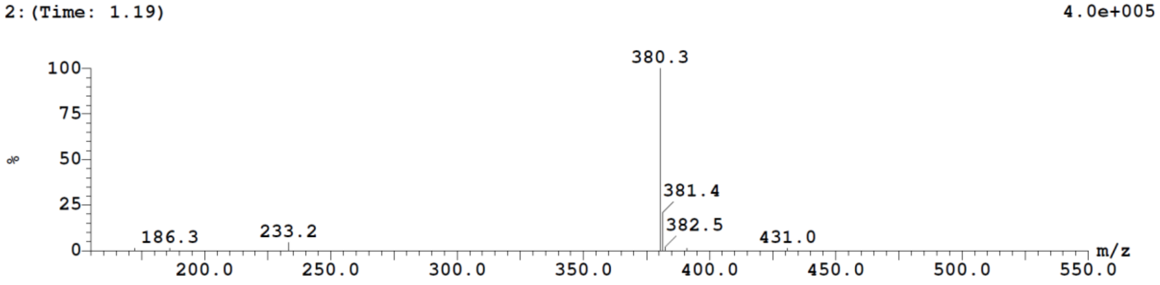

MOC-32

This report was created by ACD/NMR Processor Academic Edition. For more information go to www.acdlabs.com/nmrproc/

5/9/2017 8:40:15 AM

Acquisition Time (sec)	5.3084	***Comment***	CIX-1086668, KAU1501KTM-260_1, 1H, CD3OD - Methanol-D4 @ 231805				
Date	03 Dec 2015 21:54:24			***Date Stamp***	03 Dec 2015 21:54:24		
File Name	C:\Users\mtk0005\Google Drive\KAU\MEI-Araby\12-BIO3193-03 Imidazole Cpds\1H-NMR\KAU1501KTM-260_1\fid					***Frequency (MHz)***	300.13
Nucleus	1H	***Number of Transients***	16	***Origin***	spect	***Original Points Count***	32768
Owner	guest	***Points Count***	32768	***Pulse Sequence***	zg30	***Receiver Gain***	724.10
SW(cyclical) (Hz)	6172.84	***Solvent***	METHANOL-d4	***Spectrum Offset (Hz)***	1853.4263	***Spectrum Type***	STANDARD
Sweep Width (Hz)	6172.65	***Temperature (degree C)***	26.600				

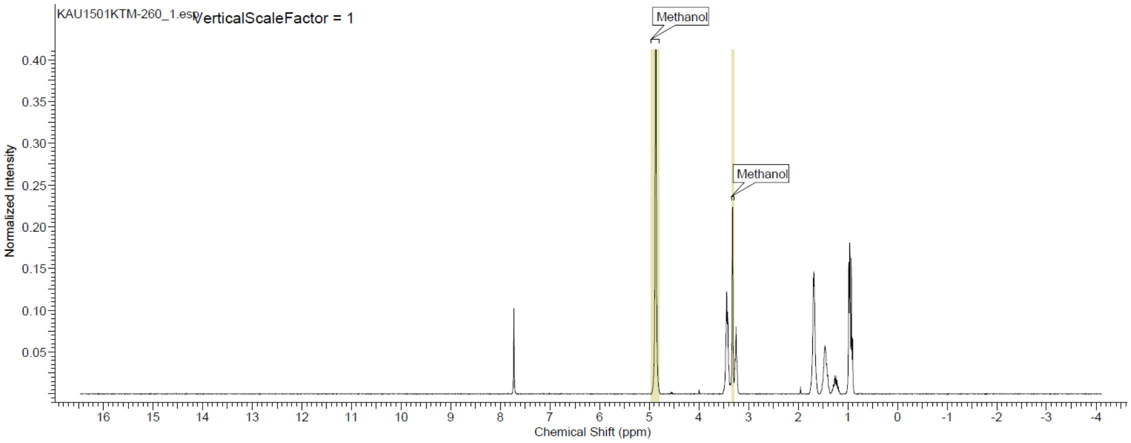

SpinWorks 4:

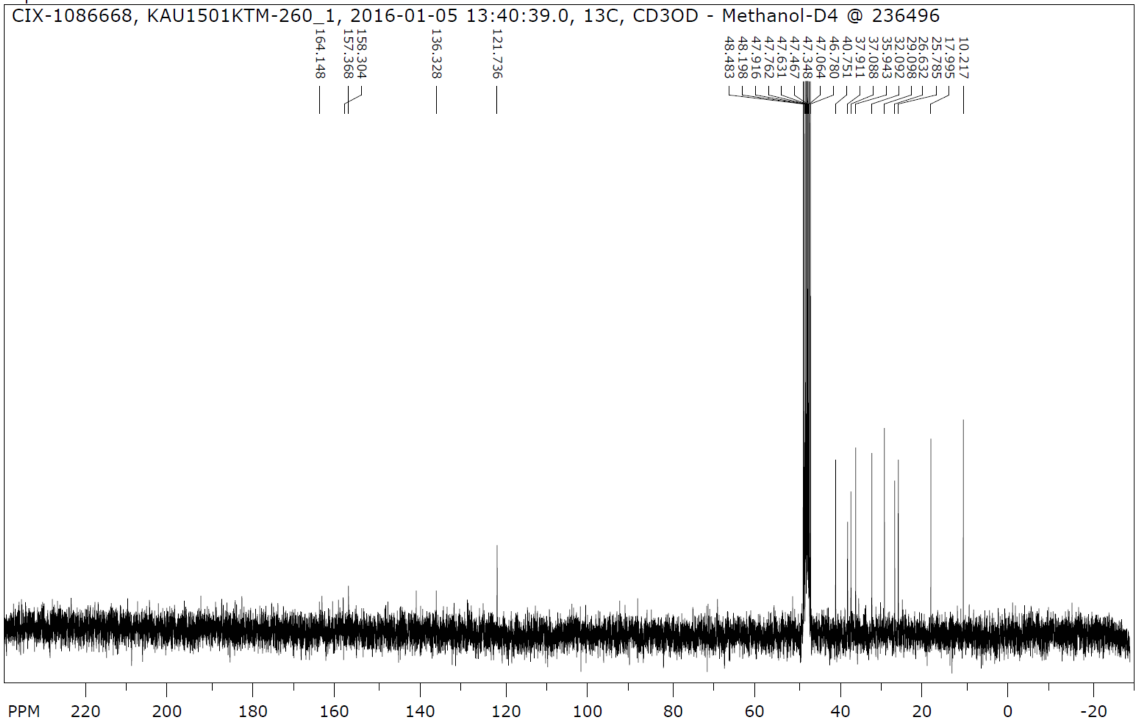

file: ...01KTM-260_1, 13C, 320 @ 236496\fid expt: <zgpg30>
transmitter freq.: 75.475670 MHz
time domain size: 65536 points
width: 20325.20 Hz = 269.2948 ppm = 0.310138 Hz/pt
number of scans: 540

freq. of 0 ppm: 75.467746 MHz
processed size: 32768 complex points
LB: 1.000 GF: 0.0000
Hz/cm: 813.008 ppm/cm: 10.77179

Peak ID	Time	Mass Found
6	1.84	351.24

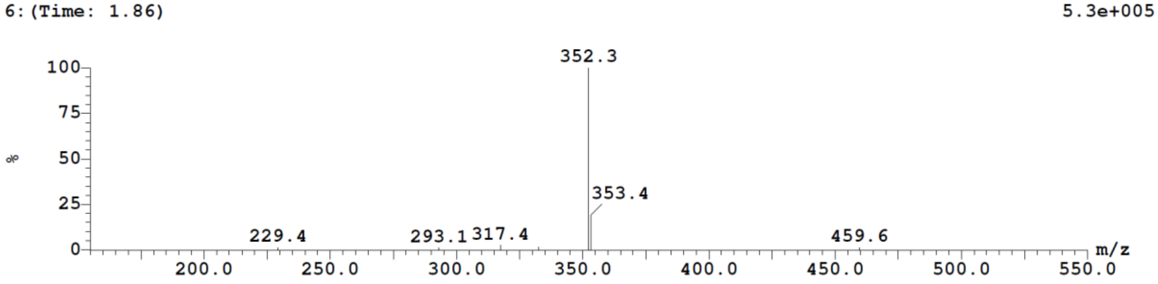

MOC-33

This report was created by ACD/NMR Processor Academic Edition. For more information go to www.acdlabs.com/nmrproc/

5/8/2017 8:06:31 AM

**Acquisition Time (sec)**	2.6477	**Comment**	CIX-1080568, KAU1501KTM-211 1, 1H, CD3OD - Methanol-D4 @ 219295				
**Date**	22 Oct 2015 11:50:56			**Date Stamp**	22 Oct 2015 11:50:56		
**File Name**	C:\Users\mtk0005\Google Drive\KAU\MEI-Araby\12-BIO3193-03 Imidazole Cpds\1H-NMR\KAU1501KTM-211 1\fid					**Frequency (MHz)**	300.13
**Nucleus**	1H	**Number of Transients**	8	**Origin**	spect	**Original Points Count**	16384
**Owner**	Administrator	**Points Count**	16384	**Pulse Sequence**	zg	**Receiver Gain**	512.00
**SW(cyclical) (Hz)**	6188.12	**Solvent**	METHANOL-d4	**Spectrum Offset (Hz)**	1853.6917	**Spectrum Type**	STANDARD
**Sweep Width (Hz)**	6187.74	**Temperature (degree C)**	24.160				

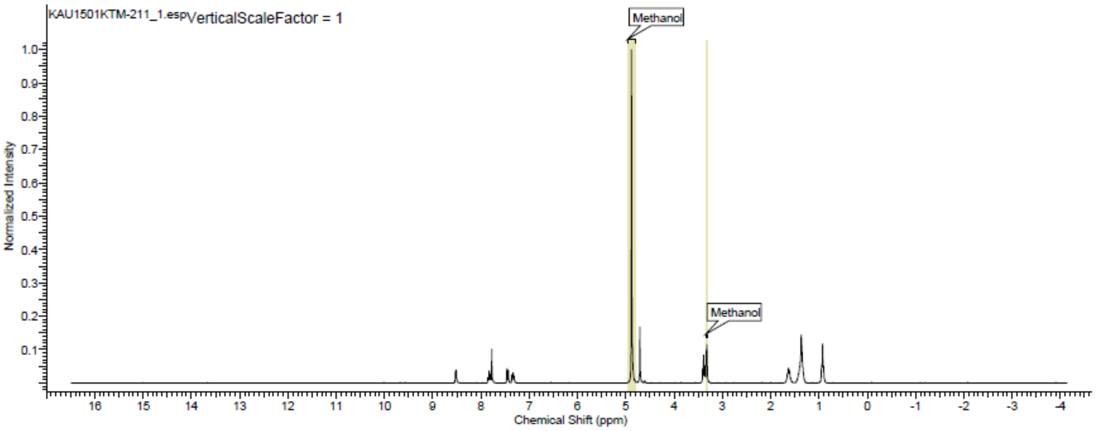

SpinWorks 4: CIX-1080568, KAU1501KTM-211_1, 2016-01-05 13:40:39.0, 13C, CD3OD - Methanol-D4 @ 236472

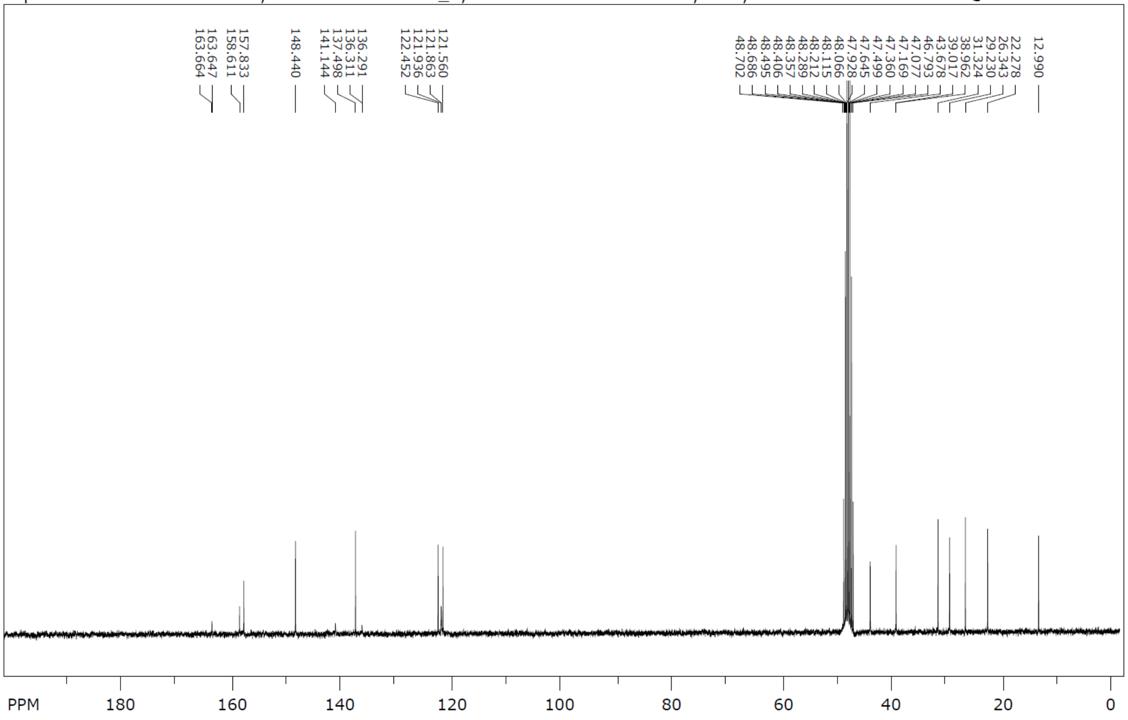

file: ...01KTM-211_1, 13C, 100 @ 236472\fid expt: <zgpg30>
transmitter freq.: 75.475670 MHz
time domain size: 65536 points
width: 20325.20 Hz = 269.2948 ppm = 0.310138 Hz/pt
number of scans: 640

freq. of 0 ppm: 75.467746 MHz
processed size: 32768 complex points
LB: 1.000 GF: 0.0000
Hz/cm: 613.961 ppm/cm: 8.13456

Peak ID	Time	Mass Found
14	2.79	329.19

14:(Time: 2.78) 3.6e+006

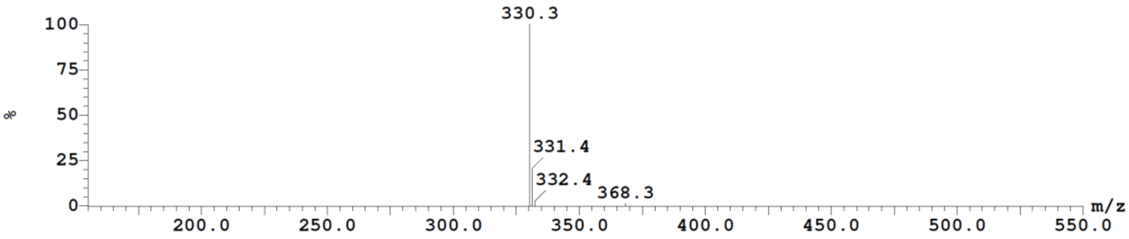

Peak ID	Time	Mass Found
7	2.50	316.21

7:(Time: 2.50) 1:MS AP+ 8.5e+005

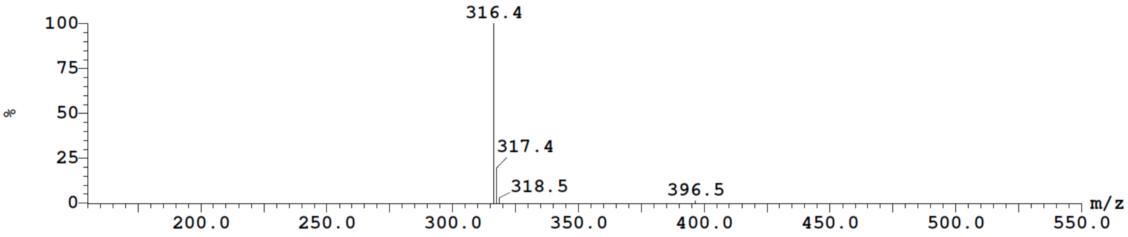

S3. Competition Assays of MOC Compounds with NS4A

Determination of $NS4A_{21`-34`}$ binding affinity

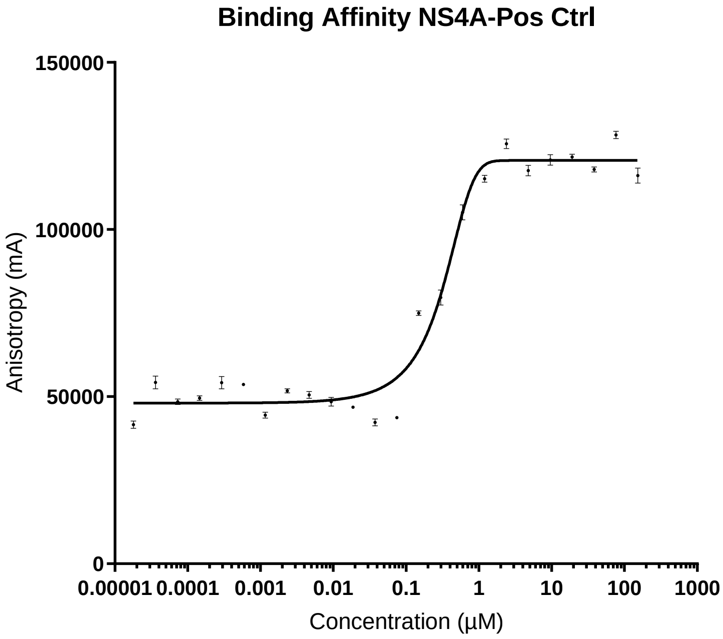

Figure S3. Fluorescence assay for binding of $NS4A_{21`-34`}$ to NS3 (Genotype 4).

S4. Molecular Dynamics

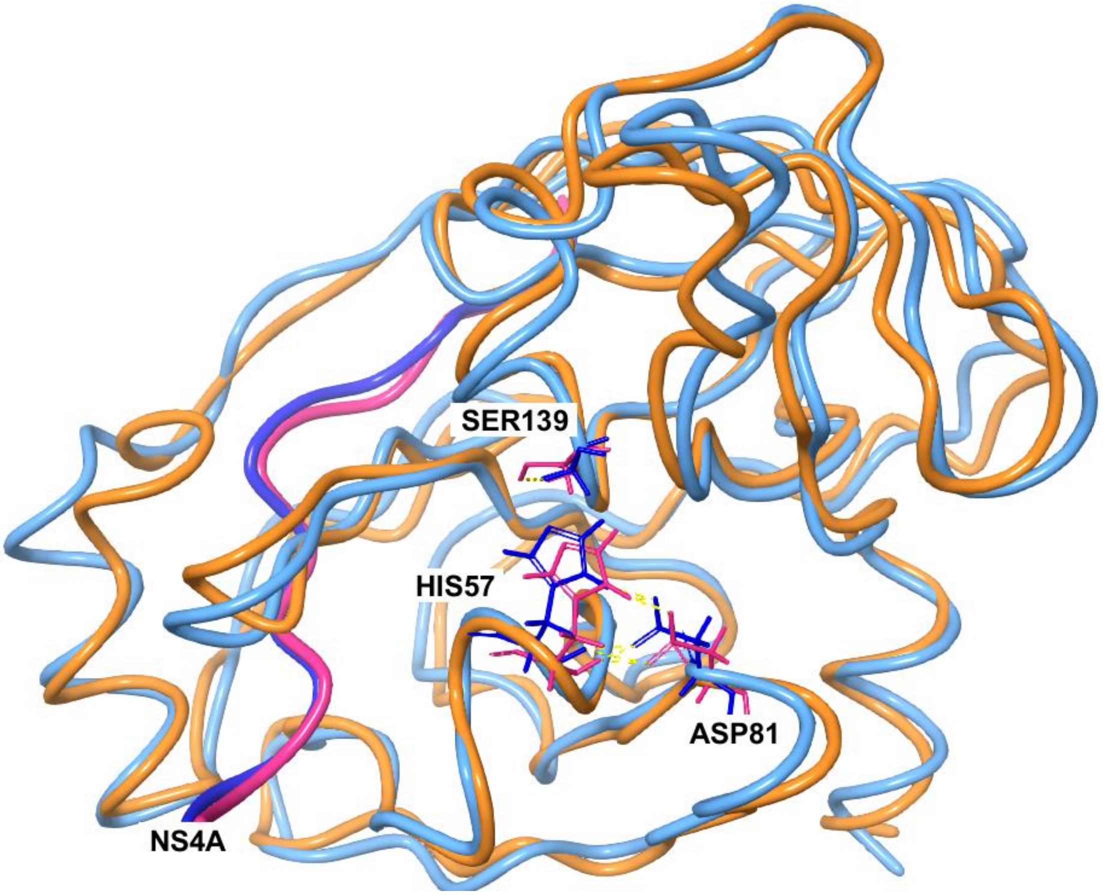

Figure S4-A: The crystal structure of Ns3 complexed with NS4A21`-32` was downloaded and prepared according to Protein Preparation protocol described in the Experimental Section 4.3.2. Note hydrogen bonding reserved in the catalytic triad His-57, Asp-81 and Ser-139.

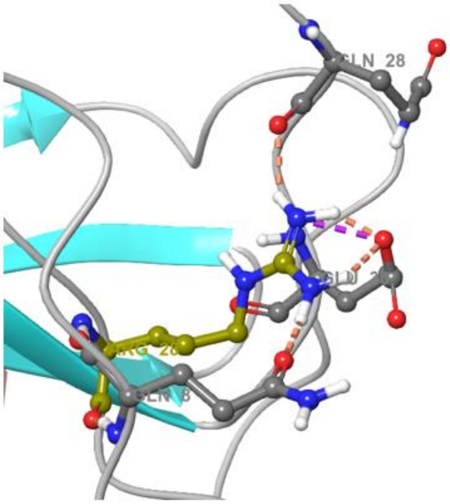

2OBO

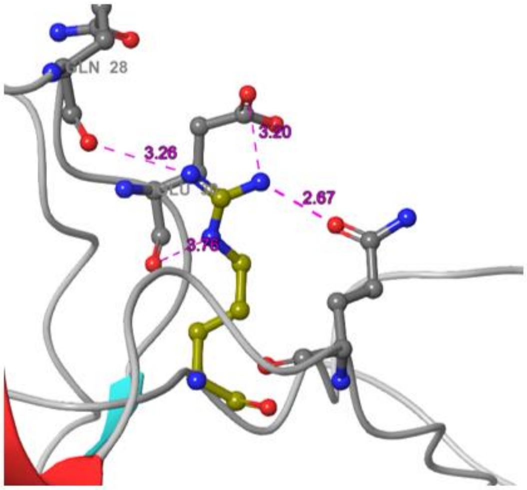

2OC1

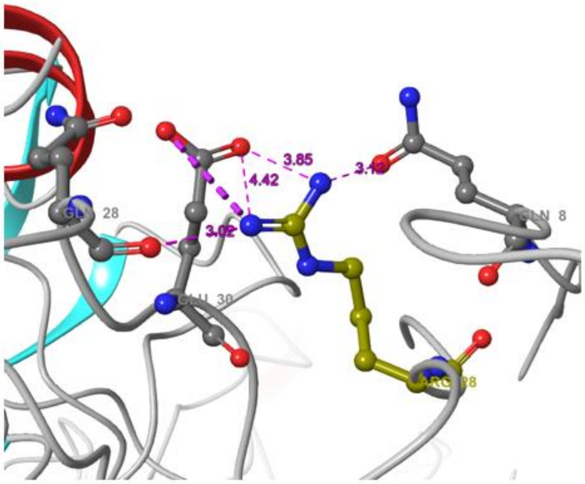

3KEE

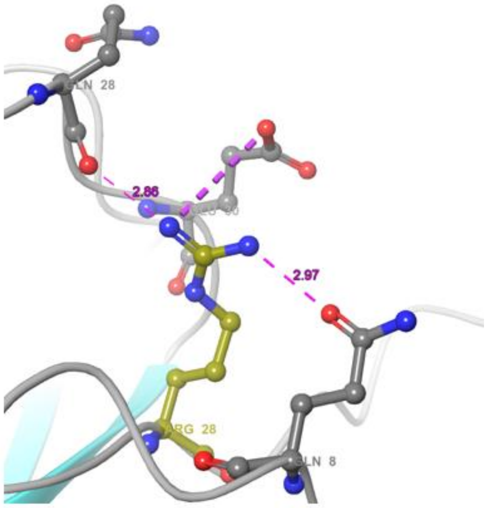

3LOX

Figure S4-B. Interactions of Arg-28` terminal guanidine at four different reported NS3/4A crystal structures (PDB code are printed under each corresponding image). Note that it commonly makes ionic interactions with Glu-30 and hydrogen bonding with Gln-8 and Gln-28 but in different conformations.

SCHRÖDINGER

Protein Secondary Structure

% Helix	% Strand	% Total SSE
9.27	32.03	41.31

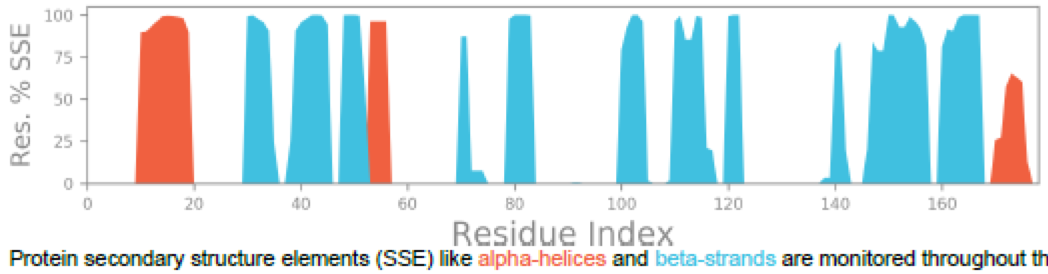

Protein secondary structure elements (SSE) like alpha-helices and beta-strands are monitored throughout the simulation. The plot above reports SSE distribution by residue index throughout the protein structure. The plot below summarizes the SSE composition for each trajectory frame over the course of the simulation, and the plot at the bottom monitors each residue and its SSE assignment over time.

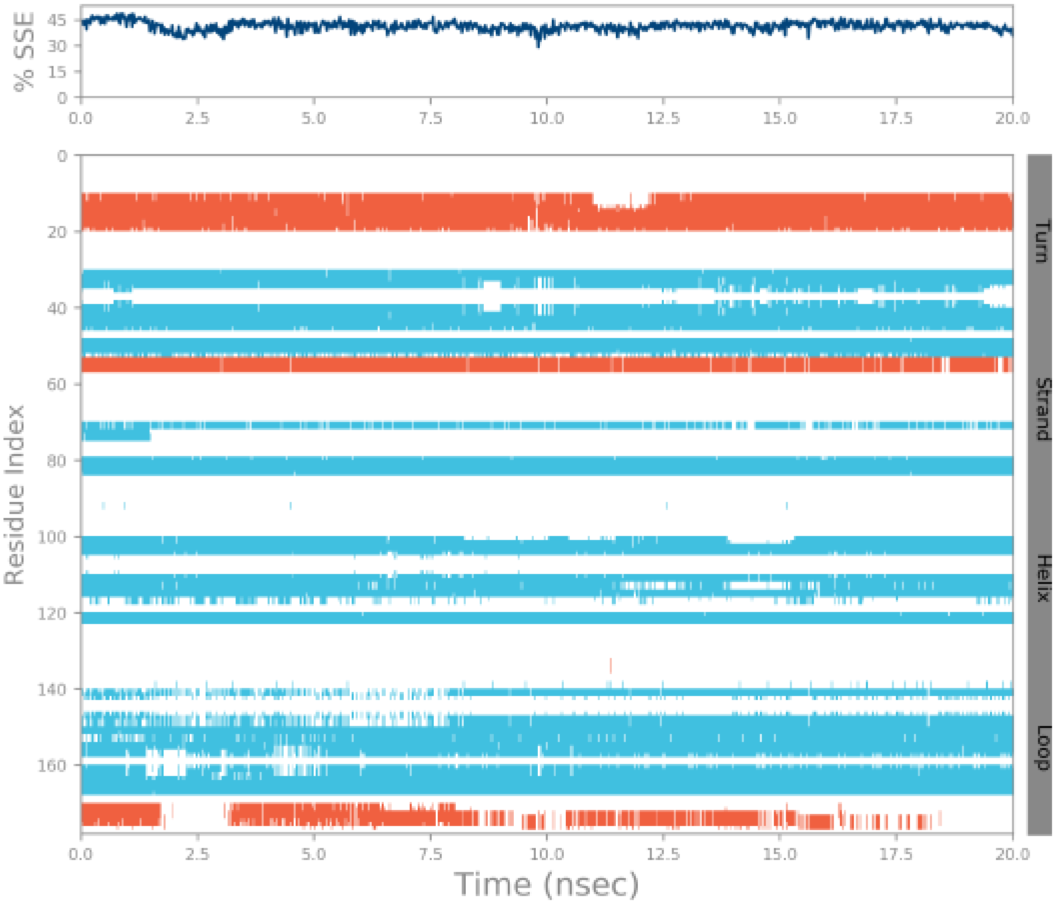

Figure S4-C. Report generated by Desmond on the secondary structure of the NS3 apoprotein during the MD simulation time. The preservation of secondary structure relates the quality of the model because a loss of seconday structure during conformational excitation would mean low quality of the binding model.
